# “Living Well” Trajectories Among Family Caregivers of People With Mild-to-Moderate Dementia in the IDEAL Cohort

**DOI:** 10.1093/geronb/gbac090

**Published:** 2022-07-07

**Authors:** Linda Clare, Laura D Gamble, Anthony Martyr, Serena Sabatini, Sharon M Nelis, Catherine Quinn, Claire Pentecost, Christina Victor, Roy W Jones, Ian R Jones, Martin Knapp, Rachael Litherland, Robin G Morris, Jennifer M Rusted, Jeanette M Thom, Rachel Collins, Catherine Henderson, Fiona E Matthews

**Affiliations:** Centre for Research in Ageing and Cognitive Health, University of Exeter Medical School, Exeter, UK; NIHR Applied Research Collaboration South-West Peninsula, Exeter, UK; Population Health Sciences Institute, Newcastle University, New Castle, UK; Centre for Research in Ageing and Cognitive Health, University of Exeter Medical School, Exeter, UK; Centre for Research in Ageing and Cognitive Health, University of Exeter Medical School, Exeter, UK; Centre for Research in Ageing and Cognitive Health, University of Exeter Medical School, Exeter, UK; Centre for Applied Dementia Studies, Bradford University, Bradford, UK; Wolfson Centre for Applied Health Research, Bradford, UK; Centre for Research in Ageing and Cognitive Health, University of Exeter Medical School, Exeter, UK; College of Health, Medicine and Life Sciences, Brunel University London, Brunel, UK; Research Institute for the Care of Older People (RICE), Bath, UK; Wales Institute for Social and Economic Research, Data and Methods, Cardiff University, Cardiff, UK; Care Policy and Evaluation Centre, London School of Economics and Political Science, London, UK; Innovations in Dementia CIC, Exeter, UK; Institute of Psychiatry, Psychology, and Neuroscience, King’s College, London, UK; School of Psychology, University of Sussex, Brighton, UK; School of Health Sciences, The University of Sydney, Sydney, Australia; Centre for Research in Ageing and Cognitive Health, University of Exeter Medical School, Exeter, UK; Care Policy and Evaluation Centre, London School of Economics and Political Science, London, UK; Population Health Sciences Institute, Newcastle University, New Castle, UK

**Keywords:** Alzheimer’s, Longitudinal, Quality of life, Satisfaction with life, Well-being

## Abstract

**Objectives:**

Understanding whether and how caregivers’ capability to “live well” changes over time, and the factors associated with change, could help target effective caregiver support.

**Methods:**

We analyzed 3 time points (12 months apart) of Improving the experience of Dementia and Enhancing Active Life (IDEAL) cohort data from coresident spouse caregivers of community-dwelling individuals who had mild-to-moderate dementia at baseline, using latent growth and growth mixture models. Capability to “live well” was derived from measures of quality of life, well-being, and satisfaction with life.

**Results:**

Data from 995 spouse caregivers at Time 1, 780 at Time 2, and 601 at Time 3 were included. The mean “living well” score decreased slightly over time. We identified 3 classes of caregivers: one with higher baseline scores declining slightly over time (Stable; 66.8%), one with low baseline scores remaining stable (Lower Stable; 26.0%), and one with higher baseline scores showing marked decline (Declining; 7.2%). Scores on baseline measures differentiated the Lower Stable, but not the Declining, from the Stable class. Longitudinally, the Declining class was associated with care recipient cognitive decline and increasing hours providing care, as well as caregiver stress and depression. Findings were similar when caregivers with other kin relationships were included.

**Discussion:**

The findings indicate the importance of prompt identification of, and support for, caregivers at risk of the declining capability to “live well” and may assist in identifying those caregivers who could benefit most from targeted support.

## Background and Objectives

Globally, there are over 55 million people living with dementia, representing an annual economic impact of more than United States $1.3 trillion (World Health Organization [WHO], 2022). Projected growth in the numbers of people living with dementia will bring major cost consequences worldwide (Prince et al., 2014). A high proportion of the costs of dementia care relate to care provided by family members (Schaller et al., 2015). It was estimated in 2015 that family members provide 82 billion hours, or 6 hours per person with dementia per day, of care. Around 71% of these hours are contributed by women, and about 40% of family caregivers are spouses or partners of the care recipient (WHO, 2022).

Understanding the experiences and needs of family caregivers and how best to support them is vital, first to enable them to sustain their role while maintaining their own health and well-being, and second, because caregiver stress has a detrimental effect on the well-being of the care recipient (Quinn et al., 2020). Caring for a family member with dementia at home is mentally and physically demanding, and these demands increase over time as the care recipient becomes more dependent. Duration of caregiving and care recipient dependence are key predictors of caregiver burden (Lindt et al., 2020). While most longitudinal studies of burden find it increases over time, a few describe stable trajectories (van den Kieboom et al., 2020). These average trajectories may mask the presence of subgroups of caregivers with varying experiences. Subgroups with different trajectories of burden have been identified (Conde-Sala et al., 2014). Poor mental health is linked to an increasing burden, especially among coresident caregivers, and subgroups with different trajectories of depressive symptoms have also been identified (Ornstein et al., 2014; Taylor Jr et al., 2008).

The complex web of factors that interact to determine why some caregivers appear more resilient than others to the demands of the role has been explored in relation to processes of stress, appraisal, and coping (Pearlin et al., 1990), the impact of caregiving on caregivers’ needs (Pini et al., 2018), and positive aspects of caregiving. Despite the demanding nature of the role, some caregivers identify positive experiences such as personal growth and deriving fulfillment from feeling they are making a difference in the life of the person with dementia, which supports their well-being (Quinn et al., 2019).

The way in which caregivers evaluate their own quality of life (QoL) can provide insight into the impact on caregivers of both positive and challenging experiences, but there is a need for more empirical evidence on factors associated with caregiver QoL (Farina et al., 2017). Modeling of cross-sectional data from the British Improving the experience of Dementia and Enhancing Active Life (IDEAL) cohort (Clare et al., 2014) demonstrated that caregivers’ psychological characteristics and health, physical fitness and health, and experiences of caregiving, both positive, such as sense of competence and coping, and negative, such as stress and social restriction, had the strongest associations with the capability to “live well,” a composite measure comprising self-ratings of QoL, well-being, and satisfaction with life (Clare et al., 2019).

While such modeling provides evidence on which to base possible approaches to better supporting caregivers, it does not account for the way in which the experience of caring at home evolves over time. Available evidence suggests relatively stable average trajectories of QoL for those continuing to care at home (Bond et al., 2003; Reed et al., 2017; Välimäki et al., 2016), but again, these average trajectories may mask the presence of subgroups with different trajectories. Understanding whether and how QoL, well-being, and satisfaction with life change over time, what factors are associated with any such changes, and whether distinct trajectories can be identified could help to target support for caregivers more effectively.

In this study, we use longitudinal data from the IDEAL cohort (Clare et al., 2014) to build on the cross-sectional model and address the following questions:

- To what extent does capability to “live well” change over 24 months for coresident spouse caregivers of people living with dementia in the community?
- Is it possible to identify subgroups of caregivers with distinct trajectories of “living well” scores?
- If so, what factors are associated with membership of these subgroups?

We hypothesized that the capability to “live well” would decline over 24 months, that it would be possible to identify subgroups with distinct trajectories, and that baseline caregiver (e.g., stress) and care recipient (e.g., dependence) factors would be associated with a decline in caregiver capability to “live well.”

## Research Design and Methods

### Design

This study presents an analysis of longitudinal data from the British IDEAL cohort (Clare et al., 2014) covering three assessment time points at 12-month intervals. Data were collected through face-to-face interviews in participants’ homes by trained interviewers. IDEAL was approved by Wales Research Ethics Committee 5 (reference 13/WA/0405) and the Ethics Committee of the School of Psychology, Bangor University (reference 11684), and is registered with UKCRN (#16593). An involvement group of people with dementia and caregivers, known as the ALWAYs (Action on Living Well: Asking You) Group, assisted with the study design and contributed to understanding the results (Litherland et al., 2018).

### Participants

This analysis focuses on the informal caregivers of community-dwelling people with dementia participating in the IDEAL cohort. The IDEAL cohort was formed by recruiting community-dwelling individuals diagnosed with mild-to-moderate dementia of any type, with a Mini-Mental State Examination (Folstein et al., 1975) score ≥ 15 on enrollment, and able to provide informed consent, from 29 National Health Service sites throughout Great Britain during 2014–2016. Where the person with dementia was willing, a family caregiver was approached to contribute as well. Caregivers provided information about the care recipient and about their own experiences. At baseline (Time 1, T1), there were 1,537 people with dementia and 1,277 caregivers. Most of the caregivers (1,035; 81%) were spouses or partners. For present purposes, first the caregivers of people who moved into residential care during the study period (*n* = 70) were excluded, followed by any caregivers who were substituted for the originally participating caregiver at T2 or T3 (*n* = 7). Of the remaining caregivers at T1, 997 were spouses or partners (hereafter “spouse caregivers”), and 206 had other relationships with the care recipients. The main analyses were conducted with coresident spouse caregivers; two caregivers who were noncohabiting partners in recently formed relationships were not included in these analyses, leaving a sample of 995 coresident spouse caregivers at T1, 780 at T2, and 601 at T3. Analyses for the whole sample, including those with other kin relationships, are presented in Supplementary Material.

### Measures

Measures are based on caregiver self-report except where indicated. See Supplementary Digital Content, Appendix 1, for additional details.

#### Demographic and clinical characteristics

Caregiver age, sex, kin relationship to the person with dementia, educational level, social class, and daily hours spent providing care, and sex and diagnosis (determined from medical records) of the person with dementia, were included in analyses.

#### Social situation

Perceived social status was assessed with the MacArthur Scale (Adler et al., 2000) and social comparison with a single bespoke question. Social isolation was assessed with the Lubben Social Network Scale (Lubben et al., 2006). U.K. Office for National Statistics measures (Office for National Statistics, 2008) were used to assess the frequency of social contact and extent of social and civic participation. Engagement in social and cultural activity was assessed with the Cultural Capital Scale (Thomson, 2004).

#### Psychological health

Depression was assessed with the Center for Epidemiologic Studies Depression Scale—Revised (Eaton et al., 2004), loneliness with the six-item De Jong-Gierveld Loneliness Scale (De Jong Gierveld & Van Tilburg, 2010), neuroticism with the mini-IPIP (Donnellan et al., 2006), which contains 20 items from the International Personality Item Pool, self-esteem with the Rosenberg Self-Esteem Scale (Rosenberg, 1965), self-efficacy with the Generalized Self-Efficacy Scale (Schwarzer & Jerusalem, 1995), and optimism with the Life Orientation Test-Revised (Scheier et al., 1994).

#### Physical health

Number of chronic conditions was assessed with the Charlson Comorbidity Index age-adjusted score (Charlson et al., 2008) and subjective health with a single question (Bowling, 2005).

#### Experiences of caregiving

Stress was assessed with the Relative Stress Scale (Greene et al., 1982). Short standardized measures assessed the role of captivity and management of meaning (Pearlin et al., 1990), social restriction (Balducci et al., 2008), and competence (Robertson et al., 2007).

#### Measures relating to the person with dementia

Caregivers rated the functional ability of the person with dementia using the Functional Activities Questionnaire (Pfeffer et al., 1982) and level of dependence with the Dependence Scale (Brickman et al., 2002), and indicated their own distress at symptoms shown by the person with dementia on the Neuropsychiatric Inventory Questionnaire (Kaufer et al., 2000). The care recipient completed the Addenbrooke’s Cognitive Examination-III (ACE-III; Hsieh et al., 2013), and the total score was included as an index of cognitive functioning.

#### Relationship quality

The Positive Affect Index (Bengtson & Schrader, 1982) was used to assess the quality of the relationship between caregiver and care recipient.

#### “Living well”

Capability to “live well” comprised measures of QoL, well-being, and satisfaction with life. The World Health Organization QoL-BREF (WHOQOL-BREF; Skevington et al., 2004) was used to measure QoL; as the measure does not yield a total score, factor analysis was conducted to estimate factor scores for those with complete data (Clare et al., 2019). Well-being was assessed with the World Health Organization-Five well-being index (WHO-5; Bech, 2004) percentage score, and satisfaction with life using the Satisfaction with Life Scale (SwLS; Diener et al., 1985).

### Modeling

Version 5 of the IDEAL data set was used. A latent “living well” factor was estimated from SwLS, WHOQOL-BREF, and WHO-5 scores and expressed on the same scale as SwLS (score range 5–35). To establish whether changes in “living well” could be considered meaningful, the Reliable Change Index (RCI; Evans et al., 1998) for WHO-5 and SwLS scores was calculated using baseline data. A change of 20.5 was considered reliable for WHO-5 and a change of 6.2 for SwLS. As WHOQOL-BREF does not yield an overall score, it was not possible to calculate an RCI for this measure.

Trajectories of “living well” over the three time points of IDEAL data collection (T1–T3) were investigated using two models in Mplus v.8.2; for additional details, see Supplementary Digital Content, Appendix 2. The first model examined mean change over time using a latent growth curve model (LGCM). The model estimates a mean intercept (mean score at baseline) and slope (mean change over time), with random effects to account for individual-level variation. The model diagram is shown in Supplementary Figure 1. The intercept loadings are fixed to 1 for each latent intercept, and 0, 1, and 2 for time based on the yearly measurement occasions. Due to only having three time points a linear trend was assumed. Associations between baseline measures and the intercept and slope of “living well” were investigated. The second model examined whether different mean trajectories of “living well” could be detected using growth mixture modeling (GMM; Jung & Wickrama, 2008). The posterior probability of class membership was used to investigate associations of baseline measures with each class through multinomial regression; odds ratios are presented with 95% confidence intervals.

Mixed-effect modeling was used to examine associations of class membership with trajectories of scores on measures assessed longitudinally. Mixed-effect modeling was conducted in R using the *lme4* package, with random effects to account for interindividual variation. Most measures had a skewed distribution, and residuals were checked for normality. A gamma distribution with a log link was fitted for most measures. A linear model was fitted for the social network, and a binomial distribution with a log link for caregiver hours (≤10 hr vs 10+ hr).

### Missing Data

Missing data for outcome measures were handled using full information maximum likelihood estimation with the assumption that data are missing at random (MAR). A sensitivity analysis was conducted for SwLS and WHO-5 to compare MAR growth mixture models with models that account for nonignorable missingness, and the MAR model was supported; further details are provided in Supplementary Digital Content, Appendix 3. Missing data for covariates were imputed using multiple imputations with chained equations in Mplus, generating 25 data sets. Estimates were combined according to Rubin’s rules.

## Results

Data from 995 coresident spouse caregivers (hereafter “caregivers”) at T1, 780 at T2, and 601 at T3 were included in analyses. Caregiver and care recipient characteristics and scores on study variables are summarized in Table 1 with additional details in Supplementary Table 1. The mean age at T1 was 72 years, and two thirds were females caring for a man with dementia. All measures except for social class, social or civic participation, management of meaning, and cognitive function of the person with dementia were associated with “living well” at baseline (Table 2). Analyses are detailed later, with additional Tables and Figures provided in Supplementary Digital Content, Appendix 4.

**Table 1.** Selected Characteristics of the Spouse Caregivers and Care Recipients Across the Three Time points

Domain	Measures	T1 (*n* = 995)	T2 (*n* = 780)	T3 (*n* = 601)
Caregiver age	Caregiver age in years (mean, *SD*, missing)	72.4 (8.3), *n* = 0	73.2 (8.0), *n* = 2	73.7 (8.0), *n* = 0
Caregiver/care recipient sex (*n*, %)	Female/male	656 (65.9%)	510 (65.4%)	388 (64.6%)
	Male/female	332 (33.4%)	263 (33.7%)	207 (34.4%)
	Female/female	6 (0.6%)	6 (0.8%)	5 (0.8%)
	Male/male	1 (0.1%)	1 (0.1%)	1 (0.2%)
Caregiver education (*n*, %)	No qualifications	249 (25.0%)	186 (23.8%)	137 (22.8%)
	School leaving certificate at 16	222 (22.3%)	180 (23.1%)	138 (23.0%)
	School leaving certificate at 18	294 (29.5%)	220 (28.2%)	172 (28.6%)
	University	226 (22.7%)	185 (23.7%)	147 (24.5%)
	Missing	4 (0.4%)	9 (1.2%)	7 (1.2%)
Caregiver social class (*n*, %)	High	441 (44.3%)	348 (44.6%)	275 (45.8%)
	Middle	389 (39.1%)	302 (38.7%)	233 (38.8%)
	Low	76 (7.6%)	57 (7.3%)	42 (7.0%)
	Missing	89 (8.9%)	73 (9.4%)	51 (8.5%)
Hours of care per day (*n*, %)	Under 1 hr	204 (20.5%)	116 (14.9%)	65 (10.8%)
	1–10 hr	356 (35.8%)	270 (34.6%)	217 (36.1%)
	10+ hr	424 (42.6%)	371 (47.6%)	312 (51.9%)
	Missing	11 (1.1%)	23 (2.9%)	7 (1.2%)
Caregiver “living well” scores	WHOQOL factor score (mean, *SD*, missing)	0.08 (2.0), *n* = 37	−0.12 (2.1), *n* = 41	−0.29 (2.1), *n* = 31
	WHO-5 (mean, *SD*, missing)	55.3 (19.7), *n* = 28	54.1 (20.3), *n* = 36	52.4 (20.2), *n* = 27
	SwLS (mean, *SD*, missing)	23.8 (6.4), *n* = 30	22.2 (6.8), *n* = 42	21.6 (6.6), *n* = 30
Care recipient diagnosis (*n*, %)	AD	564 (56.7%)	442 (56.7%)	348 (57.9%)
	VaD	103 (10.4%)	70 (9.0%)	55 (9.2%)
	Mixed AD/VaD	192 (19.3%)	164 (21.0%)	119 (19.8%)
	FTD	41 (4.1%)	34 (4.4%)	28 (4.7%)
	PDD/DLB	68 (6.8%)	53 (6.8%)	37 (6.2%)
	Unspecified/other	27 (2.7%)	17 (2.2%)	14 (2.3%)

*Notes:* AD = Alzheimer’s disease; VaD = vascular dementia; FTD = frontotemporal dementia; PDD = Parkinson’s disease dementia; DLB = dementia with Lewy bodies; WHOQOL = World Health Organization Quality of Life; WHO-5 = World Health Organization-Five well-being index; SwLS = Satisfaction with Life Scale; *SD* = standard deviation.

**Table 2.** Associations of Baseline Variables With Mean “Living Well” Score at Baseline and Over Time, and With Classes of “Living Well” for Spouse Caregivers

		LGCM: Associations of baseline measures with “living well”		GMM-CI: Associations of baseline measures with classes of “living well”	
		Association at baseline (intercept)	Association over time (slope)	Lower Stable vs Stable	Declining vs Stable
Domains	Measures	Estimate (95% CI)	Estimate (95% CI)	OR (95% CI)	OR (95% CI)
Demographic	Caregiver age	**0.05 (0.01–0.09)**	−0.01 (−0.03–0.01)	**0.96 (0.93–1.00)**	0.97 (0.93–1.02)
characteristics	Caregiver sex/person with dementia sex (ref: female/male)				
	Male/female	**2.43 (1.74–3.12)**	0.11 (−0.19–0.41)	**0.23 (0.11–0.49)**	1.07 (0.35–3.27)
	Female/female	2.19 (−1.77–6.15)	−0.70 (−2.22–0.81)	NE	7.74 (0.35–85.63)
	Male/male	−0.52 (−10.13–9.10)	−0.74 (−4.19–2.71)	NE	NE
	Caregiver education (ref: school certificate at 18)				
	No qualifications	−**1.02 (**−**1.86–**−**0.18)**	0.26 (−0.12–0.65)	**2.21 (1.02–4.79)**	0.80 (0.19–3.39)
	School certificate at 16	−0.61 (−1.48–0.26)	0.10 (−0.28–0.49)	1.81 (0.84–3.93)	0.59 (0.10–3.49)
	University	0.45 (−0.41–1.31)	−0.11 (−0.49–0.27)	1.04 (0.47–2.30)	0.41 (0.10–1.74)
	Caregiver social class (ref: high)				
	Middle	−0.42 (−1.10–0.25)	0.23 (−0.07–0.52)	0.97 (0.53–1.76)	0.90 (0.26–3.11)
	Low	−0.63 (−1.83–0.57)	0.42 (−0.13–0.98)	2.25 (0.79–6.41)	0.67 (0.02–26.82)
	Hours of care/day (ref: 10+)				
	Under 1 hr	**2.94 (2.11–3.77)**	−0.13 (−0.50–0.23)	**0.13 (0.04–0.43)**	2.50 (0.57–10.90)
	1–10 hr	**1.06 (0.36–1.76)**	−0.10 (−0.42–0.22)	**0.45 (0.24–0.84)**	2.10 (0.51–8.60)
Social situation	Social comparison	**2.30 (1.97–2.63)**	−0.10 (−0.26**–**0.05)	**0.16 (0.09–0.28)**	1.77 (0.82–3.80)
	Perceived social status	**0.99 (0.78–1.20)**	−0.08 (−0.18**–**0.01)	**0.46 (0.35–0.61)**	0.69 (0.38–1.28)
	Perceived community status	**0.70 (0.52–0.88)**	−0.02 (−0.10–0.06)	**0.60 (0.50–0.72)**	0.91 (0.67–1.23)
	Frequency of social contact	**0.21 (0.14–0.28)**	−0.03 (−0.06**–**0.00)	**0.89 (0.84–0.95)**	1.03 (0.89–1.19)
	Social network	**0.25 (0.19–0.31)**	−0.02 (−0.05**–**0.01)	**0.87 (0.81–0.92)**	1.00 (0.87–1.15)
	Cultural activity	**0.18 (0.12–0.24)**	−0.03 (−0.06**–**0.00)	**0.89 (0.84–0.94**)	0.97 (0.84–1.13)
	Civic participation (ref: none)				
	Low participation	−0.73 (−1.55**–**0.08)	0.04 (−0.31–0.40)	1.66 (0.86–3.23)	0.32 (0.03–3.68)
	High participation	−0.03 (−1.04–0.99)	−0.32 (−0.78–0.13)	0.74 (0.31–1.77)	0.90 (0.20–3.98)
	Social participation (ref: none)				
	Low participation	−0.18 (−1.07–0.71)	−0.14 (−0.54–0.26)	0.85 (0.38–1.86)	0.28 (0.01–8.57)
	High participation	0.63 (−0.09–1.35)	−0.17 (−0.48–0.14)	0.85 (0.46–1.55)	1.13 (0.32–3.99)
Psychological health	Neuroticism	−**0.92 (**−**1.02–**−**0.82)**	**0.05 (0.01–0.10)**	**1.95 (1.64–2.33)**	1.02 (0.82–1.26)
	Loneliness	−**1.40 (**−**1.56–**−**1.25)**	0.04 (−0.04–0.12)	**2.49 (1.96–3.16)**	1.04 (0.71–1.53)
	Depression	−**0.44 (**−**0.47–**−**0.40)**	**0.04 (0.02–0.06)**	**1.56 (1.25–1.95)**	0.94 (0.28–3.12)
	Self-esteem	**0.67 (0.61–0.74)**	−**0.06 (**−**0.09–**−**0.03)**	**0.51 (0.36–0.74)**	1.10 (0.83–1.67)
	Self-efficacy	**0.48 (0.40–0.55)**	−**0.06 (**−**0.09–**−**0.02)**	**0.76 (0.66–0.87)**	1.25 (0.98–1.60)
	Optimism	**0.77 (0.68–0.85)**	−**0.06 (**−**0.10–**−**0.02)**	**0.53 (0.45–0.63)**	1.15 (0.94–1.40)
Physical health	Self-rated health	**2.25 (1.98–2.52)**	−**0.15 (**−**0.28–**−**0.03)**	**0.14 (0.08–0.24)**	1.01 (0.53–1.91)
	Health conditions	−**0.32 (**−**0.45–**−**0.18)**	0.05 (−0.01–0.11)	**1.26 (1.06–1.50)**	1.03 (0.76–1.40)
Experiences of caregiving	Stress	−**0.35 (**−**0.38–**−**0.32)**	−0.00 (−0.02–0.01)	**1.30 (1.22–1.38)**	0.98 (0.90–1.07)
	Social restriction	−**1.09 (**−**1.32–**−**0.86)**	−0.01 (−0.11–0.10)	**1.89 (1.49–2.41)**	0.99 (0.62–1.60)
	Role captivity	−**1.03 (**−**1.17–**−**0.88)**	−0.03 (−0.10–0.04)	**1.80 (1.50–2.15)**	0.89 (0.62–1.28)
	Competence	**1.18 (1.00–1.36)**	−0.05 (−0.14–0.04)	**0.53 (0.43–0.66)**	1.13 (0.71–1.82)
	Management of meaning	0.01 (−0.07–0.08)	−0.01 (−0.05–0.02)	1.04 (0.98–1.11)	1.09 (0.97–1.22)
Relationship	Relationship quality	**0.54 (0.48–0.61)**	−0.02 (−0.05–0.01)	**0.72 (0.66–0.79)**	1.03 (0.78–1.35)
Care recipient diagnosis	Diagnosis (ref: AD)				
	VaD	−0.20 (−1.24–0.84)	0.34 (−0.12–0.81)	0.79 (0.35–1.81)	0.23 (0.00–5.84)
	Mixed AD/VaD	−0.27 (−1.10–0.57)	0.06 (−0.32–0.43)	1.04 (0.53–2.02)	0.56 (0.12–2.61)
	FTD	0.25 (−1.27–1.78)	0.02 (−0.65–0.69)	0.43 (0.12–1.51)	0.19 (0.00–25.31)
	PDD/DLB	−**1.76 (**−**2.99–**−**0.52)**	0.29 (−0.30–0.88)	1.77 (0.64–4.88)	1.21 (0.23–6.25)
	Unspecified/other	−1.24 (−2.84–0.37)	0.50 (−0.24–1.24)	1.33 (0.46–8.87)	NE
Care recipient measures	ACE-III	0.01 (−0.01–0.04)	**0.03 (0.01–0.05)**	1.00 (0.98–1.03)	1.01 (0.97–1.05)
	Dependence (informant)	−**0.57 (**−**0.69–**−**0.45)**	−0.02 (−0.08–0.04)	**1.44 (1.26–1.65)**	0.94 (0.76–1.17)
	FAQ (informant)	−**0.16 (**−**0.19–**−**0.12)**	−0.01 (−0.03–0.01)	**1.08 (1.04–1.12)**	0.97 (0.92–1.03)
	NP symptoms―caregiver distress	−**0.38 (**−**0.44–**−**0.33)**	0.02 (−0.00–0.05)	**1.32 (1.21–1.43)**	1.06 (0.90–1.25)

*Notes:* LGCM = latent growth curve model; GMM-CI = growth mixture model–class-invariant; OR = odds ratio; CI = confidence intervals; AD = Alzheimer’s disease; VaD = vascular dementia; FTD = frontotemporal dementia; PDD = Parkinson’s disease dementia; DLB = dementia with Lewy bodies; NP = neuropsychiatric; ACE-III = Addenbrooke’s Cognitive Examination III; Dependence = Dependence Scale; FAQ = Functional Activities Questionnaire; ref = reference; NE = not estimated. Bold indicates *p* < .05.

**Figure 1.**
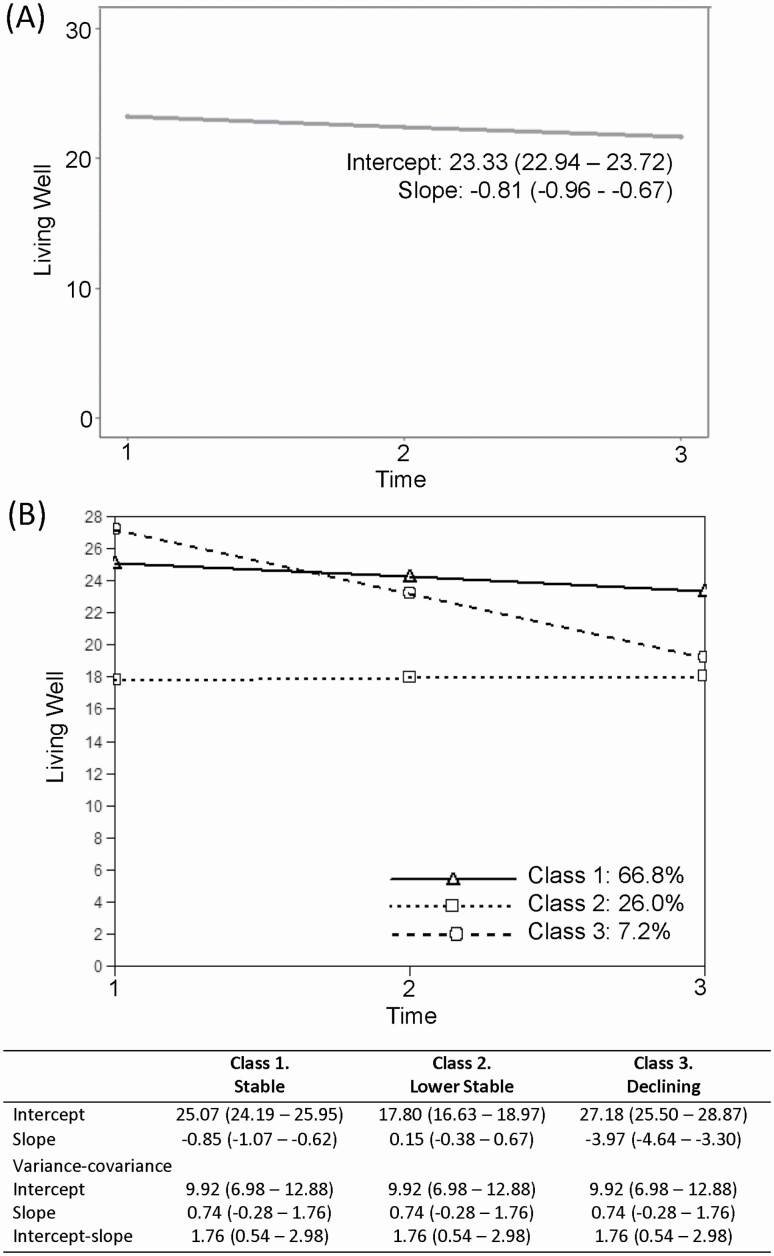
(**A**) The mean intercept and slope of “living well” for spouse caregivers determined from the latent growth curve model. (**B**) Trajectories of the three classes of “living well” of spousal caregivers, determined from the GMM-CI model; Class 1: Stable, Class 2: Lower Stable, Class 3: Declining. The mean intercepts and slopes associated with each class are shown, as are the intercept and slope variances which are equal across classes. 95% confidence intervals are displayed in brackets. GMM-CI = growth mixture model–class-invariant.

Change in “living well” score over time is summarized in Figure 1A. The LGCM fitted the data well; comparative fit index = 0.991, root mean square error of approximation = 0.043, 90% confidence interval (0.032–0.054). The mean score at baseline was 23.3, and the trajectory showed a small decrease of −0.81 units per year. Baseline scores for all psychological variables apart from loneliness, for self-rated health, and for cognitive function of the person with dementia were associated with change over time (Table 2). However, effect sizes were very small, suggesting no meaningful influence on the trajectory of “living well.”

While mean “living well” scores changed little over time, interindividual differences in the second-order growth factors were statistically significant, with estimated variances pointing to the existence of variation in both intercept and slope. We therefore investigated heterogeneity in trajectories. Model selection is described in Supplementary Tables 2–4 and Supplementary Figure 2.

The resulting three-class solution had average latent class probabilities ranging from 0.77 to 0.83 and entropy of 0.58. It comprised a class with higher baseline scores and a slight decline over time of a magnitude less than the RCI (Class 1, hereafter referred to as “Stable,” 66.8%), a stable class with lower baseline scores (Class 2, hereafter “Lower Stable,” 26.0%), and a class with initial higher scores that showed marked decline over time which could be considered a reliable change (Class 3, hereafter “Declining,” 7.2%). Trajectories alongside fixed and random effects are shown in Figure 1B, and individuals within each class are plotted in Supplementary Figure 3. Sensitivity analyses to check the assumption that data are missing at random are shown in Supplementary Table 5. Characteristics of the caregivers in each class and scores on study variables across time points are shown in Supplementary Table 6. Given some uncertainty in class membership, further analyses took into account the probabilities of each individual being a member of each class.

Associations of baseline measures with class membership were examined using multinomial regression with the Stable class as the reference group and are summarized in Table 2. The Lower Stable class showed clear differences, with members more likely to be women caring for men, to be younger, to have no educational qualifications, and to be providing more hours of care compared to the Stable class. They were more likely to have poorer baseline scores on all measures except the management of meaning and social and civic participation, and to be caring for people with poorer functional ability and higher levels of dependence. The Declining class was similar to the Stable class at baseline, and there were no significant differences that could explain the reasons for the decline. Further analyses explored whether decline might be explained by changes over time in the condition of the care recipient; findings are summarized in Table 3. Compared to the Stable class, care recipients in the Declining class were likely to have a greater decline in cognition and to require more hours of care over time, with caregivers likely to experience increasing distress over time in response to neuropsychiatric symptoms and to report poorer relationship quality. Caregivers in the Declining class were also more likely to experience increased depression, stress and role captivity, and declining subjective health and competence compared to the Stable class. Despite those in the Lower Stable class being more likely to have higher levels of stress at baseline compared to those in the Stable class, stress was more likely to increase over time for those in the Stable class, while remaining high for those in the Lower Stable class, with similar findings seen for dependence and functional impairment.

**Table 3.** Associations of “Living Well” With Longitudinal Study Measures, Where Available, for Spouse Caregivers

		Associations of longitudinal measures with classes of “living well”	
Domains	Measures	Lower Stable vs Stable	Declining vs Stable
Generalized linear mixed model		OR (95% CI)	OR (95% CI)
Characteristics	Hours of care/day (10+ vs ≤10)	1.04 (0.50–2.18)	**5.28 (1.65–16.90)**
Psychological health	Depression	0.88 (0.76–1.02)	**1.82 (1.41–2.34)**
Physical health	Self-rated health	0.99 (0.94–1.05)	**0.85 (0.79–0.92)**
	Health conditions	0.99 (0.94–1.05)	1.01 (0.91–1.12)
Experiences of caregiving	Stress	**0.87 (0.80–0.93)**	**1.47 (1.29–1.67)**
	Social restriction	1.00 (0.94–1.06)	1.06 (0.95–1.18)
	Role captivity	0.94 (0.89–1.00)	**1.26 (1.15–1.39)**
	Competence	1.02 (0.98–1.05)	**0.90 (0.84–0.95)**
	Management of meaning	0.99 (0.96–1.03)	1.01 (0.95–1.06)
Relationship	Relationship quality	0.99 (0.95–1.03)	**0.89 (0.84–0.95)**
Care recipient measures	ACE-III	0.97 (0.88–1.09)	**0.94 (0.87–0.99)**
	Dependence (informant)	**0.87 (0.81–0.95)**	1.03 (0.89–1.19)
	FAQ (informant)	**0.85 (0.77–0.95)**	1.12 (0.94–1.34)
	NP symptoms―caregiver distress	0.93 (0.79–1.10)	**1.84 (1.38–2.45)**
Linear mixed model		Estimate (95% CI)	Estimate (95% CI)
Social situation	Social network	−0.09 (−0.67–0.48)	−**1.26 (**−**2.20**–−**0.31)**

*Notes:* OR = odds ratio; CI = confidence intervals; NP = neuropsychiatric; ACE-III = Addenbrooke’s Cognitive Examination III; Dependence = Dependence Scale; FAQ = Functional Activities Questionnaire. Bold indicates *p* < .05.

The analyses incorporating the full sample of caregivers are provided in Supplementary Digital Content, Appendix 5. These analyses produced similar classes and patterns of baseline associations; for details see Supplementary Tables 7–10 and Supplementary Figures 4 and 5.

## Discussion and Implications

This is one of the relatively few studies offering a longitudinal perspective on QoL, well-being, and satisfaction with life of family caregivers of people with dementia, and to the best of our knowledge, the first to identify groups with different “living well” trajectories. Focusing on coresident spouse caregivers, results from our large cohort indicated a generally stable trajectory over 24 months, with a negligible yearly decline in a combined “living well” score, and did not support the hypothesis of decline over time. Further analysis did, as hypothesized, yield subgroups, and three groups were identified with Stable, Lower Stable, and Declining “living well” scores. The hypothesis that baseline caregiver and care recipient factors would be associated with the decline was not supported; while baseline scores on most measures differentiated the Lower Stable from the Stable group, no baseline variables differentiated the Declining group from the Stable group. However, longitudinal decline in “living well” scores were associated with increasing cognitive impairment in the care recipient, the impact of neuropsychiatric symptoms, and hours of care provided.

A stable or only slightly declining pattern was seen in over 90% of the sample, and this finding is consistent with earlier reports of QoL trajectories over 18–36 months (Bond et al., 2003; Reed et al., 2017; Välimäki et al., 2016) and WHO-5 scores at 12-month follow-up (Kurten et al., 2021). However, 24 months can be a relatively short period in the overall duration of care provision for many caregivers, and so even a small annual decline of the degree seen in the Stable group could potentially amount to a meaningful change over a longer period. One third of the cohort had low but stable “living well” scores. Among factors associated with lower scores were poorer psychological and physical health, social situation, relationship quality, and experiences of caregiving, confirming previous findings about the relevance of these factors (Clare et al., 2019; Farina et al., 2017; Fauth et al., 2012).

The proportion identified as having a declining trajectory, although small, is not negligible. Nothing distinguished the Declining class at baseline, but changes over time in the needs of the care recipient were associated with changes in caregivers’ psychological and physical health, experiences of caregiving, and scores on measures of “living well.” Our findings are consistent with the observation that increased supervision time predicted increased caregiver burden at 3-month follow-up (Lethin et al., 2020) and a decline in caregiver WHO-5 scores at 12-month follow-up (Kurten et al., 2021).

The study has several limitations. As might be expected in a sample of older people, there was considerable attrition in the cohort and some of this attrition could have been selective. For example, those with lower “living well” scores may have been more likely to withdraw from the study at the next time point. However, alternate growth mixture models were explored, which take into account nonignorable dropout for two of the measures used to estimate the “living well” score. As class formation and estimates of the intercepts and slopes were almost identical to those found with the model where data are assumed to be missing at random, there was no evidence of selective attrition based on “living well” scores. With data from three-time points available, linear trends had to be assumed, whereas in reality, patterns might be more complex (Fauth et al., 2012). The cohort included a relatively high proportion of spouse caregivers, leading to the decision to focus the main analyses on this group. While this has the advantage of yielding a homogeneous sample, the needs of caregivers with other kin relationships may differ somewhat and are important to consider. The sample was mainly White British, reflecting population norms and the profile of memory clinic attenders (Pham et al., 2018). While this again has the advantage of providing a homogeneous group, the findings cannot be assumed to generalize straightforwardly to other ethnic groups or cultures. The care recipients had mild-to-moderate dementia at baseline, which may have limited the extent of variation in caregivers’ experiences, and while the proportions with rarer dementia subtypes were in line with population estimates (Prince et al., 2014), actual numbers were small. In addition, the classes extracted from the GMM-class invariant model should be interpreted with some caution, as GMM is an exploratory approach and findings vary based on model specification. While a GMM with free variances both within and across classes is optimal, to support convergence it was necessary to constrain the intercept and slope variances to be equal across classes. However, plots of the resulting classes show clear distinctions in the patterns of trajectories. Despite these limitations, analyses were based on a large and well-described sample and incorporated a wide range of relevant variables, suggesting the findings are likely to be robust.

The key implication of our findings for public policy is that failing to provide accessible, practical support for family caregivers may be a false economy. In the UK, the policy of fiscal austerity that has dominated public services since the 2008 financial crisis has resulted in a marked reduction in availability of publicly funded social care for people with dementia, such as home care, day care, and respite services. Linked to this, an increase in provision of informal care, and in particular in the proportion of caregivers providing care for 20+ hours per week, has been identified (Zigante et al., 2021). This has implications for social care services and for health services; where informal care arrangements become strained or break down completely, this can result in costly, unnecessary hospitalization or institutionalization. The findings also have implications for research and practice. Alongside practical support, the availability of evidence-based approaches that can support the well-being and mental health of family caregivers, and reduce a subjective sense of burden, is important. A recent systematic review (Wiegelmann et al., 2021) concluded that while cognitive-behavioral interventions appeared helpful for supporting mental health and leisure or physical activity interventions for reducing subjective burden, it was not possible to reach a general conclusion about which types of intervention are most effective. One key reason for this was that few attempts had been made to target subgroups of caregivers defined according to their characteristics or level of need or risk, and hence the authors concluded that future research on caregiver support should adopt a more targeted approach. Our findings also suggest that support should be differentially targeted. While all caregivers may benefit from programs designed to equip them to cope well, different approaches may be required for those with higher and lower levels of well-being. Furthermore, there is a need for prompt identification of caregivers at risk of the declining capability to “live well” so that appropriate support can be offered at key transition points as the needs of the care recipient increase. Future research may address these challenges.

In conclusion, this study, one of few providing a longitudinal perspective on QoL, well-being, and satisfaction with life among family caregivers of people with dementia, adds to an understanding of caregivers’ experiences and needs. It demonstrates for the first time that differing trajectories underlie relative overall stability in mean scores on these measures of “living well.” Over two thirds had higher initial scores that remained relatively stable, but some declined over time as the needs and dependence of the care recipient increased, and one quarter had markedly low “living well” scores from the outset. The findings highlight the importance of providing accessible, practical support for family caregivers as a matter of public policy, understanding how to target supportive interventions appropriately, and developing the capability to promptly identify and support caregivers who are at high risk of decline in well-being.

## Supplementary Material

gbac090_suppl_Supplementary_Material

## References

[CIT0001] Adler, N. E., Epel, E. S., Castellazzo, G., & Ickovics, J. R. (2000). Relationship of subjective and objective social status with psychological and physiological functioning: Preliminary data in healthy white women. Health Psychology, 19(6), 586–592. doi:10.1037/0278-6133.19.6.586 11129362

[CIT0002] Balducci, C., Mnich, E., McKee, K. J., Lamura, G., Beckmann, A., Krevers, B., Wojszel, Z. B., Nolan, M., Prouskas, C., Bien, B., & Oberg, B. (2008). Negative impact and positive value in caregiving: Validation of the COPE index in a six-country sample of carers. The Gerontologist, 48(3), 276–286. doi:10.1093/geront/48.3.276 18591353

[CIT0003] Bech, P . (2004). Measuring the dimension of psychological general well-being by the WHO-5. Quality of Life Newsletter, 32(1), 15–16.

[CIT0004] Bengtson, V. L., & Schrader, S. S. (1982). Parent–child relations. In D. J. Mangon & W. A. Peterson (Eds.), Research instruments in social gerontology: Social roles and social participation (Vol. 2, pp. 115–185). University of Minnesota Press.

[CIT0005] Bond, M. J., Clark, M. S., & Davies, S. (2003). The quality of life of spouse dementia caregivers: Changes associated with yielding to formal care and widowhood. Social Science and Medicine, 57(12), 2385–2395. doi:10.1016/S0277-9536(03)00133-3 14572845

[CIT0006] Bowling, A . (2005). Just one question: If one question works, why ask several? Journal of Epidemiology and Community Health, 59(5), 342–345. doi:10.1136/jech.2004.021204 15831678 PMC1733095

[CIT0007] Brickman, A. M., Riba, A., Bell, K., Marder, K., Albert, M., Brandt, J., & Stern, Y. (2002). Longitudinal assessment of patient dependence in Alzheimer disease. Archives of Neurology, 59(8), 1304–1308. doi:10.1001/archneur.59.8.1304 12164728

[CIT0008] Charlson, M. E., Charlson, R. E., Peterson, J. C., Marinopoulos, S. S., Briggs, W. M., & Hollenberg, J. P. (2008). The Charlson comorbidity index is adapted to predict costs of chronic disease in primary care patients. Journal of Clinical Epidemiology, 61(12), 1234–1240. doi:10.1016/j.jclinepi.2008.01.006 18619805

[CIT0009] Clare, L., Nelis, S. M., Quinn, C., Martyr, A., Henderson, C., Hindle, J. V., Jones, I. R., Jones, R. W., Knapp, M., Kopelman, M. D., Morris, R. G., Pickett, J. A., Rusted, J. M., Savitch, N. M., Thom, J. M., & Victor, C. R. (2014). Improving the experience of dementia and enhancing active life―living well with dementia: Study protocol for the IDEAL study. Health and Quality of Life Outcomes, 12(1), 164. doi:10.1186/s12955-014-0164-6 25433373 PMC4260182

[CIT0010] Clare, L., Wu, Y.-T., Quinn, C., Jones, I. R., Victor, C. R., Nelis, S. M., Martyr, A., Litherland, R., Pickett, J. A., Hindle, J. V., Jones, R. W., Knapp, M., Kopelman, M. D., Morris, R. G., Rusted, J. M., Thom, J. M., Lamont, R. A., Henderson, C., Rippon, I., … Matthews, F. E.; on behalf of the IDEAL study team (2019). A comprehensive model of factors associated with capability to “live well” for family caregivers of people living with mild-to-moderate dementia: Findings from the IDEAL study. Alzheimer Disease and Associated Disorders, 33(1), 29–35. doi:10.1097/WAD.0000000000000285 30802226 PMC6416095

[CIT0011] Conde-Sala, J. L., Turró-Garriga, O., Calvó-Perxas, L., Vilalta-Franch, J., Lopez-Pousa, S., & Garre-Olmo, J. (2014). Three-year trajectories of caregiver burden in Alzheimer’s disease. Journal of Alzheimer’s Disease, 42(2), 623–633. doi:10.3233/JAD-140360 24919767

[CIT0012] De Jong Gierveld, J., & Van Tilburg, T. (2010). The De Jong Gierveld short scales for emotional and social loneliness: Tested on data from 7 countries in the UN generations and gender surveys. European Journal of Ageing, 7(2), 121–130. doi:10.1007/s10433-010-0144-6 20730083 PMC2921057

[CIT0013] Diener, E., Emmons, R. A., Larsen, R. J., & Griffin, S. (1985). The satisfaction with life scale. Journal of Personality Assessment, 49(1), 71–75. doi:10.1207/s15327752jpa4901_13 16367493

[CIT0014] Donnellan, M. B., Oswald, F. L., Baird, B. M., & Lucas, R. E. (2006). The mini-IPIP scales: Tiny-yet-effective measures of the Big Five factors of personality. Psychological Assessment, 18(2), 192–203. doi:10.1037/1040-3590.18.2.192 16768595

[CIT0015] Eaton, W. W., Smith, C., Ybarra, M., Muntaner, C., & Tien, A. (2004). Center for Epidemiologic Studies Depression Scale: Review and revision (CESD and CESD-R). In M. E. Maruish (Ed.), The use of psychological testing for treatment planning and outcomes assessment (3rd ed., Vol. 3: Instruments for Adults, pp. 363–377). Lawrence Erlbaum.

[CIT0016] Evans, C., Margison, F., & Barkham, M. (1998). The contribution of reliable and clinically significant change methods to evidence-based mental health. Evidence Based Mental Health, 1(3), 70–72. doi:10.1136/ebmh.1.3.70

[CIT0017] Farina, N., Page, T. E., Daley, S., Brown, A., Bowling, A., Basset, T., Livingston, G., Knapp, M., Murray, J., & Banerjee, S. (2017). Factors associated with the quality of life of family carers of people with dementia: A systematic review. Alzheimer’s and *Dementia*, 13(5), 572–581. doi:10.1016/j.jalz.2016.12.010 28167069

[CIT0018] Fauth, E., Hess, K., Piercy, K., Norton, M., Corcoran, C., Rabins, P., Lyketsos, C., & Tschanz, J. (2012). Caregivers’ relationship closeness with the person with dementia predicts both positive and negative outcomes for caregivers’ physical health and psychological well-being. Aging and Mental Health, 16(6), 699–711. doi:10.1080/13607863.2012.678482 22548375 PMC3430821

[CIT0019] Folstein, M. F., Folstein, S. E., & McHugh, P. R. (1975). “Mini-mental state”. A practical method for grading the cognitive state of patients for the clinician. Journal of Psychiatric Research, 12(3), 189–198. doi:10.1016/0022-3956(75)90026-6 1202204

[CIT0020] Greene, J. G., Smith, R., Gardiner, M., & Timbury, G. C. (1982). Measuring behavioural disturbance of elderly demented patients in the community and its effects on relatives: A factor analytic study. Age and Ageing, 11(2), 121–126. doi:10.1093/ageing/11.2.121 7102472

[CIT0021] Hsieh, S., Schubert, S., Hoon, C., Mioshi, E., & Hodges, J. R. (2013). Validation of the Addenbrooke’s Cognitive Examination III in frontotemporal dementia and Alzheimer’s disease. Dementia and Geriatric Cognitive Disorders, 36(3–4), 242–250. doi:10.1159/000351671 23949210

[CIT0022] Jung, T., & Wickrama, K. A. S. (2008). An introduction to latent class growth analysis and growth mixture modeling. Social and Personality Psychology Compass, 2(1), 302–317. doi:10.1111/j.1751-9004.2007.00054.x

[CIT0023] Kaufer, D. I., Cummings, J. L., Ketchel, P., Smith, V., MacMillan, A., Shelley, T., Lopez, O. L., & DeKosky, S. T. (2000). Validation of the NPI-Q, a brief clinical form of the Neuropsychiatric Inventory. The Journal of Neuropsychiatry and Clinical Neurosciences, 12(2), 233–239. doi:10.1176/jnp.12.2.233 11001602

[CIT0024] Kurten, L., Dietzel, N., Kolominsky-Rabas, P. L., & Graessel, E. (2021). Predictors of the one-year-change in depressiveness in informal caregivers of community-dwelling people with dementia. BMC Psychiatry, 21(1), 177. doi:10.1186/s12888-021-03164-8 33812389 PMC8019174

[CIT0025] Lethin, C., Leino-Kilpi, H., Bleijlevens, M. H., Stephan, A., Martin, M. S., Nilsson, K., Nilsson, C., Zabalegui, A., & Karlsson, S. (2020). Predicting caregiver burden in informal carers caring for persons with dementia living at home: A follow-up cohort study. Dementia, 19(3), 640–660. doi:10.1177/1471301218782502 29929383

[CIT0026] Lindt, N., van Berkel, J., & Mulder, B. C. (2020). Determinants of overburdening among informal carers: A systematic review. BMC Geriatrics, 20(1), 304. doi:10.1186/s12877-020-01708-3 32847493 PMC7448315

[CIT0027] Litherland, R., Burton, J., Cheeseman, M., Campbell, D., Hawkins, M., Hawkins, T., Oliver, K., Scott, D., Ward, J., Nelis, S. M., Quinn, C., Victor, C., & Clare, L. (2018). Reflections on PPI from the “Action on Living Well: Asking You” advisory network of people with dementia and carers as part of the IDEAL study. Dementia, 17(8), 1035–1044. doi:10.1177/1471301218789309 30373457

[CIT0028] Lubben, J., Blozik, E., Gillmann, G., Iliffe, S., von Renteln Kruse, W., Beck, J. C., & Stuck, A. E. (2006). Performance of an abbreviated version of the Lubben Social Network Scale among three European community-dwelling older adult populations. The Gerontologist, 46(4), 503–513. doi:10.1093/geront/46.4.503 16921004

[CIT0029] Office for National Statistics. (2008). Harmonised concepts and questions for social data sources, secondary standards. Social capital. Office for National Statistics.

[CIT0030] Ornstein, K., Gaugler, J. E., Zahodne, L., & Stern, Y. (2014). The heterogeneous course of depressive symptoms for the dementia caregiver. The International Journal of Aging and Human Development, 78(2), 133–148. doi:10.2190/AG.78.2.c 24956922 PMC4240506

[CIT0031] Pearlin, L. I., Mullan, J. T., Semple, S. J., & Skaff, M. M. (1990). Caregiving and the stress process: An overview of concepts and their measures. The Gerontologist, 30(5), 583–594. doi:10.1093/geront/30.5.583 2276631

[CIT0032] Pfeffer, R. I., Kurosaki, T. T., Harrah, C. H. Jr. Chance, J. M., & Filos, S. (1982). Measurement of functional activities in older adults in the community. Journal of Gerontology, 37(3), 323–329. doi:10.1093/geronj/37.3.323 7069156

[CIT0033] Pham, T. M., Petersen, I., Walters, K., Raine, R., Manthorpe, J., Mukadam, N., & Cooper, C. (2018). Trends in dementia diagnosis rates in UK ethnic groups: Analysis of UK primary care data. Clinical Epidemiology, 10(1), 949–960. doi:10.2147/CLEP.S152647 30123007 PMC6087031

[CIT0034] Pini, S., Ingleson, E., Megson, M., Clare, L., Wright, P., & Oyebode, J. R. (2018). A needs-led framework for understanding the impact of caring for a family member with dementia. The Gerontologist, 58(2), e68–e77. doi:10.1093/geront/gnx148 29562360 PMC5946854

[CIT0035] Prince, M., Knapp, M., Guerchet, M., McCrone, P., Prina, M., Comas-Herrera, A., Wittenberg, R., Adelaja, B., Hu, B., & King, D. (2014). Dementia UK: Second edition―Overview. Alzheimer’s Society.

[CIT0036] Quinn, C., Nelis, S. M., Martyr, A., Morris, R. G., Victor, C., & Clare, L.; on behalf of the IDEAL study team (2020). Caregiver influences on “living well” for people with dementia: Findings from the IDEAL study. Aging and Mental Health, 24(9), 1505–1513. doi:10.1080/13607863.2019.1602590 31104475

[CIT0037] Quinn, C., Nelis, S. M., Martyr, A., Victor, C., Morris, R. G., & Clare, L.; on behalf of the IDEAL study team (2019). Influence of positive and negative dimensions of dementia caregiving on caregiver well-being and satisfaction with life: Findings from the IDEAL study. The American Journal of Geriatric Psychiatry, 27(8), 838–848. doi:10.1016/j.jagp.2019.02.005 30917903

[CIT0038] Reed, C., Barrett, A., Lebrec, J., Dodel, R., Jones, R. W., Vellas, B., Wimo, A., Argimon, J. M., Bruno, G., & Haro, J. M. (2017). How useful is the EQ-5D in assessing the impact of caring for people with Alzheimer’s disease? Health and Quality of Life Outcomes, 15(1), 16. doi:10.1186/s12955-017-0591-2 28109287 PMC5251250

[CIT0039] Robertson, S. M., Zarit, S. H., Duncan, L. G., Rovine, M. J., & Femia, E. E. (2007). Family caregivers’ patterns of positive and negative affect. Family Relations, 56(1), 12–23. doi:10.1111/j.1741-3729.2007.00436.x

[CIT0040] Rosenberg, M . (1965). Society and the adolescent self-image. Princeton University Press.

[CIT0041] Scheier, M. F., Carver, C. S., & Bridges, M. W. (1994). Distinguishing optimism from neuroticism (and trait anxiety, self-mastery, and self-esteem): A reevaluation of the Life Orientation Test. Journal of Personality and Social Psychology, 67(6), 1063–1078. doi:10.1037/0022-3514.67.6.1063 7815302

[CIT0051] Schaller, S., Mauskopf, J., Kriza, C., Wahlster, P., & Kolominsky-Rabas, P. L. (2015). The main cost drivers in dementia: a systematic review. International Journal of Geriatric Psychiatry, 30(2), 111–129. doi:10.1002/gps.4198 25320002

[CIT0042] Schwarzer, R., & Jerusalem, M. (1995). Generalized self-efficacy scale. In J. Weinman, S. Wright, & M. Johnston (Eds.), Measures in health psychology: A user’s portfolio. causal and control beliefs (pp. 35–37). NFER-NELSON.

[CIT0043] Skevington, S. M., Lotfy, M., & O’Connell, K. A.; WHOQOL Group (2004). The World Health Organization’s WHOQOL-BREF quality of life assessment: Psychometric properties and results of the international field trial. A report from the WHOQOL group. Quality of Life Research, 13(2), 299–310. doi:10.1023/B:QURE.0000018486.91360.00 15085902

[CIT0044] Taylor Jr, D. H., Ezell, M., Kuchibhatla, M., Østbye, T., & Clipp, E. C. (2008). Identifying trajectories of depressive symptoms for women caring for their husbands with dementia. Journal of the American Geriatrics Society, 56(2), 322–327. doi:10.1111/j.1532-5415.2007.01558.x 18179488 PMC3679900

[CIT0045] Thomson, K . (2004). Cultural capital and social exclusion survey: Technical report. National Centre for Social Research.

[CIT0046] Välimäki, T. H., Martikainen, J. A., Hongisto, K., Väätäinen, S., Sintonen, H., & Koivisto, A. M. (2016). Impact of Alzheimer’s disease on the family caregiver’s long-term quality of life: Results from an ALSOVA follow-up study. Quality of Life Research, 25(3), 687–697. doi:10.1007/s11136-015-1100-x 26350541

[CIT0047] van den Kieboom, R., Snaphaan, L., Mark, R., & Bongers, I. (2020). The trajectory of caregiver burden and risk factors in dementia progression: A systematic review. Journal of Alzheimer’s Disease, 77(3), 1107–1115. doi:10.3233/jad-200647 PMC7683084 32804093

[CIT0048] Wiegelmann, H., Speller, S., Verhaert, L.-M., Schirra-Weirich, L., & Wolf-Ostermann, K. (2021). Psychosocial interventions to support the mental health of informal caregivers of persons living with dementia: A systematic literature review. BMC Geriatrics, 21(1), 94. doi:10.1186/s12877-021-02020-4 33526012 PMC7849618

[CIT0049] World Health Organization (2022). Global status report on the public health response to dementia. World Health Organization.

[CIT0050] Zigante, V., Fernandez, J.-L., & Mazzotta, F. (2021). Changes in the balance between formal and informal care supply in England between 2001 and 2011: Evidence from census data. Health Economics, Policy and Law, 16(2), 232–249. doi:10.1017/S1744133120000146 32611466

