# Supplementary material for: “Living Well” Trajectories Among Family Caregivers of People With Mild-to-Moderate Dementia in the IDEAL Cohort
Source: J Gerontol B Psychol Sci Soc Sci. 2022 Jul 7;77(10):1852–63. doi: 10.1093/geronb/gbac090 (PMC9535787; doi:10.1093/geronb/gbac090)
Supplement: gbac090_suppl_Supplementary_Material [file gbac090_suppl_supplementary_material.pdf]

'Living well' trajectories among family caregivers of people with mild-to-moderate dementia in the IDEAL cohort
SUPPLEMENTARY MATERIAL

Linda Clare ScD[1,2], Laura D. Gamble PhD[3], Anthony Martyr PhD[1], Serena Sabatini PhD[1], Sharon M. Nelis PhD[1], Catherine Quinn PhD[4,5], Claire Pentecost PhD[1], Christina Victor PhD[6], Roy W Jones MBBS[7], Ian R. Jones PhD[8], Martin Knapp PhD[9], Rachael Litherland MSc[10], Robin G. Morris PhD[11], Jennifer M. Rusted PhD[12], Jeanette M. Thom PhD[13], Rachel Collins PhD[1], Catherine Henderson PhD[9], and Fiona E. Matthews PhD[3], on behalf of the IDEAL study team

1. Centre for Research in Ageing and Cognitive Health, University of Exeter Medical School, UK
2. NIHR Applied Research Collaboration South-West Peninsula, Exeter, UK
3. Population Health Sciences Institute, Newcastle University, UK
4. Centre for Applied Dementia Studies, Bradford University, UK
5. Wolfson Centre for Applied Health Research, Bradford, UK
6. College of Health, Medicine and Life Sciences, Brunel University London, UK
7. Research Institute for the Care of Older People (RICE), Bath, UK
8. Wales Institute for Social and Economic Research, Data and Methods, Cardiff University, UK
9. Care Policy and Evaluation Centre, London School of Economics and Political Science, UK
10. Innovations in Dementia CIC, Exeter, UK
11. Institute of Psychiatry, Psychology and Neuroscience, King's College London, UK
12. School of Psychology, University of Sussex, Brighton, UK
13. School of Health Sciences, The University of Sydney, Sydney, Australia

Appendix 1. Further details of measures included in the analyses

Except where otherwise indicated, all measures relate to the caregiver and are based on caregiver self-report.

Demographic and clinical c*haracteristics*. Demographic information was collected by interviewers including caregiver age, sex, relationship to the care recipient (spouse/partner or family/friend), whether the caregiver is co-resident with the care recipient, education (no qualifications; school leaving certificate at 16; school leaving certificate at 18; college/university), and social class (I/II, high; III-NM/III-M, middle; IV/V/armed forces, low; Office for National Statistics, 2010). The number of hours spent caring per day was collected and categorized into under 1 hour, 1-10 hours, and over 10 hours. Sex of the person with dementia was recorded. Dementia diagnosis was obtained from the medical records of the person with dementia and classified into seven groups; Alzheimer's disease (AD); vascular dementia (VaD); mixed AD/VaD; frontotemporal dementia (FTD); Parkinson's disease dementia (PDD); dementia with Lewy bodies (DLB); unspecified/other. For analysis purposes, PDD and DLB subtypes were combined. Where the specific diagnosis changed over the course of the study, the last recorded diagnosis was used; this applied to 28 participants.

Social situation. The MacArthur Scale of Subjective Social Status (Adler et al., 2000) was used to assess perceived standing in society and in the community, with participants asked to place themselves on a ladder ranging from 1 (low) to 10 (high). Social comparison was measured with a single question 'Do you think compared to most other people your age, your situation is….' with responses ranging from much worse to much better. Social isolation was measured using the six-item Lubben Social Network Scale (score range 0-30; Lubben et al., 2006); higher scores indicate more social contact. To assess social capital, several measures are provided by the UK Office for National Statistics (Office for National Statistics, 2008). Frequency of social contact was measured using a 9-item measure of contact with friends, relatives, and neighbors, with a higher score indicating more contact (scale 0-32). Civic participation was a single item question 'In the last 12

months have you taken any of the following actions in an attempt to solve a problem affecting people in your area' with a list of seven actions responses which are summed to provide a score out of 7. Social participation is assessed in terms of involvement in unpaid help to groups, using the question 'During the last 12 months have you given any unpaid help to any groups, clubs, or organizations in the ways listed below', with a list of 12 responses which are summed to provide a score out of 12. For both civic and social participation, higher scores indicate more participation. For analyses, scores were categorized into no participation (score of 0), low participation (score of 1), and high participation (score >1). Engagement in social activity was measured with the thirteen-item Cultural Capital Scale; higher scores indicate greater engagement (score range 13-65; Thomson, 2004).

Psychological health. The Center for Epidemiologic Studies Depression Scale-Revised (CESD-R) was used to measure depression (score range 0-60), with higher scores indicative of more depressive symptoms (Eaton et al., 2004). Loneliness was measured using the six-item De Jong-Gierveld Loneliness Scale (score range 0-6; De Jong Gierveld & Van Tilburg, 2010); higher scores indicate greater loneliness. Neuroticism was measured using the mini-IPIP neuroticism measure, where a higher score indicates higher rates of neuroticism (score range 4-20; Donnellan et al., 2006). Self-esteem was measured using the ten-item Rosenberg Self-Esteem Scale (score range 10-40; Rosenberg, 1965); higher scores indicate greater self-esteem. Perceived self-efficacy was measured using the general sense of perceived efficacy scale (score range 10-40), with higher scores indicative of better self-efficacy (Schwarzer & Jerusalem, 1995). The six non-filler items from the Life Orientation Test-Revised scale (Scheier et al., 1994) were used to measure optimism (score range 0-24); higher scores indicate greater optimism.

Physical health. The Charlson Comorbidity Index (CCI) age-adjusted score (Charlson et al., 2008; Charlson et al., 1987) identified the number of chronic conditions. Subjective health was assessed with the question "How would you rate your health in the past four weeks?" with six ordinal response options ranging from very poor to excellent (Bowling, 2005).

Experiences of caregiving. The Relative Stress Scale is a 15-item measure assessing the degree of distress and social upset experienced by a relative as a result of caring for a person with physical or behavioral difficulties (score range 0-60); a higher score indicates more severe stress (Greene et al., 1982). Role captivity (score range 0-12) is measured using three items assessing the extent that caregivers feel trapped in their role; higher scores are worse (Pearlin et al., 1990). Management of Meaning is a 9-item measure assessing the extent that caregivers of people with dementia feel they have lost aspects of their personality due of caring (score range 9-36; a higher score is worse; Pearlin et al., 1990); higher scores are worse. The Modified Social Restriction Scale (MSRS) contains two items asking how easy it is to find someone to look after the care recipient if the caregiver needs a break or is unwell (Balducci et al., 2008); a higher score indicates more difficulties. Caregiver competency is measured using three items assessing the extent to which caregivers of people with dementia feel they are doing an adequate job as a caregiver (Robertson et al., 2007); possible scores range from 0-12, with higher scores indicating greater competence.

Measures relating to the person with dementia. Caregivers used the modified 11-item Functional Activities Questionnaire (FAQ; Martyr et al., 2012; Pfeffer et al., 1982) to rate the current functional ability of the care recipient (score range 0-33) and the Dependence Scale (Brickman et al., 2002), a 13-item questionnaire measuring the amount of assistance needed by the person with dementia, to rate dependence (score range 0-15). In both cases, a higher score indicates greater functional impairment. Caregivers rated their level of emotional distress in response to specific symptoms they identifed as experienced by the person with dementia on the Neuropsychiatric Inventory Questionnaire (score range 0-35; Kaufer et al., 2000; Morris & National Alzheimer's Coordinating Center, 2008); higher scores indicate higher distress. People with dementia were administered the Addenbrooke's Cognitive Examination-III (ACE-III; Hsieh et al., 2013) and the total score was used as a measure of cognitive ability. Scores ranged from 0-100, with higher scores indicating better cognition.

Relationship quality. The Positive Affect Index provided a measure of current relationship quality (Bengtson & Schrader, 1982). Responses to five questions on a 6-point scale are summed for a total score (range 5-30); a higher score indicates better relationship quality.

'Living well'. Caregivers' capability to 'live well' was defined using three individual self-rated measures covering caregivers' quality of life, well-being, and satisfaction with life. Quality of life was measured with the World Health Organization Quality of Life-BREF (WHOQOL-BREF), which is designed to measure multiple components related to quality of life (Skevington et al., 2004). It includes two single indicators (overall quality of life and general health) and four domains (physical health, psychological health, social relationships, and environment). There is no total score, and to create one a factor analysis was conducted to estimate factor scores for those with complete data as previously described (Clare et al., 2019; Wu et al., 2021). Well-being was measured by the World Health Organization-Five Well-being Index (WHO-5; score range 0-100), which includes items on positive mood, vitality, and general interests (Bech, 2004). Satisfaction with life was measured using the Satisfaction with Life Scale (SwLS; score range 5-35), which is designed to measure global judgements of satisfaction with life (Diener et al., 1985).

Appendix 2. Further details of the statistical modelling undertaken

Latent Growth Curve Model

To determine how 'living well' changes over time for caregivers of people with dementia, latent growth curve modelling (LGCM) was conducted in Mplus Version 8.2 (Muthén & Muthén, 1998-2017) using the first three timepoints of IDEAL data (T1-T3). The LGCM comprised a measurement model which was then extended to a second order growth model allowing estimation of the mean intercept (baseline) and the mean slope (change over time) of 'living well', with random effects to account for variation across individuals (Wickrama et al., 2016).

The measurement model involves building the latent factor 'living well' from measures of SwLS, WHOQOL-BREF, and WHO-5 at each year by longitudinal confirmatory factor analysis (LCFA). For WHOQOL-BREF factor scores were generated as previously described (Clare et al., 2019; Wu et al., 2021). SwLS was selected as the marker variable with loading fixed to 1 at each timepoint, and the intercept fixed to zero to allow for model identification. The scale of 'living well' took on the same scale as SwLS and the variance of each latent factor and covariance among latent factors were defined by SwLS (Brown, 2006). The associations among WHOQOL-BREF and WHO-5 were estimated relative to their association with SwLS. Variances were estimated for each subdomain indicator and autocorrelated errors specified and retained in the model to avoid misspecification (Little, 2013).

A good model will have a Comparative Fit index (CFI) and Tucker-Lewis index (TLI) greater than 0.90, and a root mean square error of approximation (RMSEA) less than 0.08 (Hu & Bentler, 1999). As shown in Supplementary Table 2, the unconstrained measurement model (configural model) was a good fit to the data indicating that each factor is defined by the same variables and that the same general pattern of factor loadings hold across time (Millsap & Cham, 2012; Millsap & Olivera-Aguilar, 2012). In order for meaningful comparisons to be made in a LCFA, the assumption of longitudinal measurement invariance should be met (Byrne & Watkins,

2003; Chen, 2007). Three levels of measurement invariance were tested imposing additional restrictions at each step: metric invariance (constrained factor loadings across measurement occasions), scalar invariance (constrained factor loadings across measurement occasions and intercepts across time to be equal), and strict invariance (constrained factor loadings across measurement occasions, and intercepts and residual variances across time to be equal). Each level of measurement invariance was applied, and a range of model fit indices examined to ensure that the model fit did not weaken when each level of constraint was applied. Studies have suggested that CFI, RMSEA, and standardized root mean squared residual (SRMR) are the most important indicators when it comes to testing measurement invariance, with cut offs of <0.01 change in CFI, <0.015 change in RMSEA, and <0.030 change in SRMR (Chen, 2007; Cheung & Rensvold, 2002). ΔX^2 was also examined but is sensitive to sample size so was not relied upon (Chen, 2007; Kline, 2011; Schermelleh-Engel et al., 2003). As shown in Supplementary Table 2, the changes within the model fit indices for each more constricted model were within these limits supporting the view that metric, scalar, and strict measurement invariance held. Further analyses were conducted with the strict invariance model.

The ‘living well’ factors defined in the LCFA model were used as indicators of the second-order growth curve where the intercept and slope global factors of ‘living well’ are estimated, each with their associated mean and variance. The model diagram is shown in Supplementary Figure 1. The intercept loadings were fixed to 1 for each latent intercept, and 0, 1, and 2 for time based on the yearly measurement occasions. Due to only having 3 timepoints a linear trend was assumed.

Latent class growth analysis and growth mixture modelling

So far, our approach assumes growth trajectories of all individuals can be adequately described using a single estimate of growth parameters. We employed latent class growth analysis (LCGA) and growth mixture modelling (GMM) to examine whether multiple growth trajectories of ‘living well’ exist in the IDEAL caregiver population (Jung & Wickrama, 2008; Muthén, 2004;

Muthén & Shedden, 1999). Different assumptions were tested. The LCGA fixes variances of the global growth factors to zero across classes (assuming trajectories within a class are homogeneous). The GMM-CI (class-invariant) constrains the variances of the global growth factors across classes to be equal, and the GMM-CV (class-varying) freely estimates all variances of the global growth factors across classes. A frequent problem with these models is non-convergence and local solutions (Hipp & Bauer, 2006), with the more complex models, particularly those with free variances, more likely to experience convergence difficulties (Grimm & Ram, 2009). Between 1 and 5 class solutions were tested for each assumption, with 1,000 random starts and 20 iterations for each model in order to avoid local solutions. Following successful convergence, the optimal number of distinct trajectories was determined using Bayesian Information Criterion (BIC), sample size adjusted BIC (ssBIC), the Lo-Mendell-Rubin likelihood ratio test (LMR-LRT), and the bootstrapped likelihood ratio test (BLRT) which provide between model comparisons (k vs k-1), and entropy (Jung & Wickrama, 2008; Nylund et al., 2007; Tein et al., 2013). Entropy is a standardized index of a model-based classification accuracy based on the average posterior probability, with higher values indicating clearer class separation (Muthén, 2004). Substantive criteria were based on a class size greater than 1% and theoretical and practical interpretability of the classes. Upon finding an optimal solution, the model was repeated with double the number of starts and iterations to ensure a global solution.

Due to their person-centered approach, GMMs allow for examination of predictors of class membership (Wickrama et al., 2016). The categorical latent class is related to the covariates by way of multinomial logistic regression which assigns each individual fractionally to all classes using posterior probabilities. Predictors of class were examined using the ‘3-step’ approach in Mplus (R3STEP) in order to protect the latent class structure from influences of the covariates (Asparouhov & Muthen, 2013; Vermunt, 2010).

Latent Trajectory Model selection

For the cohort of co-resident spouse caregivers, three models were tested as described above: LCGA, GMM-CI and GMM-CV. One to 5 class solutions of each model were conducted, and results are displayed in Supplementary Table 3 for those models that converged, along with the associated plots in Supplementary Figure 2. The LCGA and GMM-CI models are less computationally intensive, and all classes ran with no convergence issues. The GMM-CV model with free intercepts and slopes experienced convergence issues. For the full sample of caregivers (spouse and family/friend), the LCGA and GMM-CI models also ran with no issues, with the GMM-CV model failing to converge. Given all available information including model fit indices, interpretability, and theoretical considerations, the 3-class solution was selected for both co-resident spouse caregivers and all caregivers.

Appendix 3. Sensitivity analysis to explore non-ignorable missing data models

Models that account for non-ignorable dropout

Growth modelling based on maximum-likelihood estimation tends to assume that data are missing at random (MAR; Little & Rubin, 2002). MAR is suitable when dropout is predicted by covariates or observed outcome values but if dropout does not fulfil the MAR assumption, missing data techniques that handle non-ignorable missingness may be more appropriate. Sensitivity analyses were conducted to compare the 3-class MAR growth mixture model for two of the measures used to estimate 'living well', SwLS, and WHO-5, with two non-ignorable dropout models described by Muthén and colleagues; Roy latent dropout pattern mixture models and Beunckens selection models (Muthén et al., 2011). The Roy latent dropout pattern-mixture model divides participants into groups based on their dropout pattern (Roy, 2003), and the Beunckens selection model combines the linear model for the observed responses with a logistic regression model for the non-ignorable dropout process (Beunckens et al., 2008).

Comparison of results

Three-class growth mixture models were explored for SwLS and WHO-5. As shown in Supplementary Table 5, the Roy pattern-mixture model and the Beunckens selection model produced almost identical classes with similar estimates for the intercepts and slopes as the MAR model. Because results from all three approaches agree, the MAR model is supported and this model is suitable for the analysis.

Appendix 4. Supplementary details of analyses for spouse caregivers

Supplementary Figure 1. The latent growth curve model involves the measurement model where the factors living well T1 – living well T3 are determined from measures of WHOQOL-BREF, WHO-5, and SwLS of spousal caregivers at T1-T3. SwLS is used as the marker variable and loading fixed to 1. Intercept and slope latent factors are modelled from 'living well', with intercepts fixed to 1, and the occasions of the slopes fixed to 0, 1, and 2.

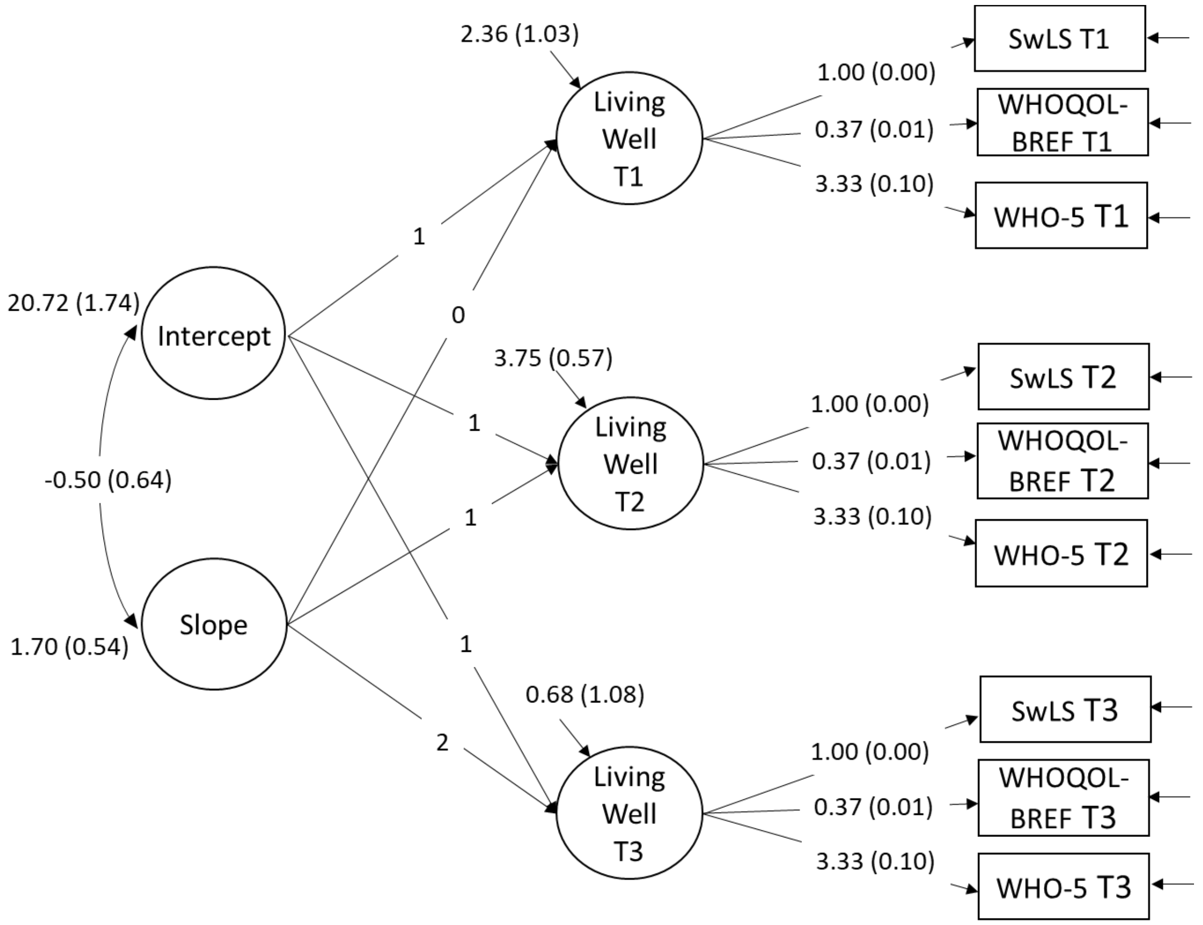

Supplementary Table 1. Characteristics of spouse caregivers and care recipients, and scores on study variables, across the three timepoints

Domain	Measures	T1 (n=995)	T2 (n=780)	T3 (n=601)
CAREGIVER				
'Living well'	WHOQOL Environment (mean, sd, missing)	16.2 (2.0), n=29	16.0 (2.0), n=39	15.9 (2.0), n=27
	WHOQOL Physical (mean, sd, missing)	15.1 (3.0), n=28	14.9 (2.9), n=37	14.7 (2.9), n=30
	WHOQOL Psychological (mean, sd, missing)	14.9 (2.2), n=30	14.7 (2.2), n=37	14.5 (2.3), n=27
	WHOQOL Social relations (mean, sd, missing)	14.7 (2.6), n=32	14.5 (2.6), n=39	14.3 (2.7), n=29
	WHOQOL Quality of Life (mean, sd, missing)	3.8 (0.8), n=23	3.7 (0.8), n=35	3.6 (0.8), n=25
	WHOQOL Health (mean, sd, missing)	3.4 (1.0), n=22	3.5 (1.0), n=35	3.4 (1.0), n=25
	WHOQOL factor score (mean, sd, missing)	0.08 (2.0), n=37	-0.12 (2.1), n=41	-0.29 (2.1), n=31
	WHO-5 (mean, sd, missing)	55.3 (19.7), n=28	54.1 (20.3), n=36	52.4 (20.2), n=27
	SwLS (mean, sd, missing)	23.8 (6.4), n=30	22.2 (6.8), n=42	21.6 (6.6), n=30
Demographic characteristics	Caregiver age in years (mean, sd)	72.4 (8.3)	73.2 (8.0)	73.7 (8.0)
	Caregiver/care recipient sex (n, %)			
	Female/male	656 (65.9%)	510 (65.4%)	388 (64.6%)
	Male/female	332 (33.4%)	263 (33.7%)	207 (34.4%)
	Female/female	6 (0.6%)	6 (0.8%)	5 (0.8%)
	Male/male	1 (0.1%)	1 (0.1%)	1 (0.2%)
	Caregiver education (n, %)			
	No qualifications	249 (25.0%)	186 (23.8%)	137 (22.8%)
	School leaving certificate at 16	222 (22.3%)	180 (23.1%)	138 (23.0%)
	School leaving certificate at 18	294 (29.5%)	220 (28.2%)	172 (28.6%)
	University	226 (22.7%)	185 (23.7%)	147 (24.5%)
	Missing	4 (0.4%)	9 (1.2%)	7 (1.2%)
	Caregiver social class (n, %)			
	High	441 (44.3%)	348 (44.6%)	275 (45.8%)
	Middle	389 (39.1%)	302 (38.7%)	233 (38.8%)
	Low	76 (7.6%)	57 (7.3%)	42 (7.0%)
	Missing	89 (8.9%)	73 (9.4%)	51 (8.5%)

	Hours of care provided per day (n, %)			
	Under 1 hour	204 (20.5%)	116 (14.9%)	65 (10.8%)
	1-10 hours	356 (35.8%)	270 (34.6%)	217 (36.1%)
	10+ hours	424 (42.6%)	371 (47.6%)	312 (51.9%)
	Missing	11 (1.1%)	23 (2.9%)	7 (1.2%)
Psychological health	Neuroticism (mean, sd, missing)	10.8 (3.1), n=28		
	Loneliness (mean, sd, missing)	2.5 (1.9), n=68	-	2.9 (1.9), n=36
	Depression (mean, sd, missing)	7.0 (7.6), n=63	8.1 (8.8), n=65	8.7 (9.2), n=46
	Self-esteem (mean, sd, missing)	31.2 (4.3), n=47		
	Self-efficacy (mean, sd, missing)	31.6 (4.1), n=44		
	Optimism (mean, sd, missing)	14.7 (3.5), n=36		
Physical health	Self-rated health (mean, sd, missing)	4.0 (1.1), n=12	3.9 (1.1), n=32	3.8 (1.0), n=19
	Health conditions (mean, sd, missing)	5.5 (2.6), n=107	5.2 (1.9), n=74	5.3 (1.9), n=50
Experiences of caregiving	Stress (mean, sd, missing)	19.0 (9.7), n=62	21.9 (10.1), n=48	23.3 (10.2), n=39
	Social restriction (mean, sd, missing)	3.5 (1.4), n=38	3.8 (1.4), n=46	3.9 (1.4), n=30
	Role captivity (mean, sd, missing)	5.4 (2.2), n=34	5.9 (2.5), n=43	6.1 (2.6), n=27
	Competence (mean, sd, missing)	9.2 (1.7), n=32	9.1 (1.6), n=45	9.0 (1.5), n=27
	Management of meaning (mean, sd, missing)	25.9 (4.5), n=54	26.3 (4.5), n=60	26.0 (4.3), n=43
Social situation	Frequency of social contact (mean, sd, missing)	19.3 (4.9), n=107		
	Social network (mean, sd, missing)	17.7 (5.5), n=36	17.3 (5.2), n=42	17.0 (5.3), n=34
	Cultural activity (mean, sd, missing)	24.6 (5.3), n=72		
	Civic participation (n, %)			
	No participation	658 (66.1%)		
	Low participation	181 (18.2%)		
	High participation	103 (10.4%)		
	Missing	53 (5.3%)		
	Social participation (n, %)			
	No participation	515 (51.8%)		
	Low participation	153 (15.4%)		
	High participation	271 (27.2%)		
	Missing	56 (5.6%)		
	Social comparison (mean, sd, missing)	3.4 (1.0), n=13		
	Standing in society (mean, sd, missing)	6.6 (1.5), n=41		

	Standing in the community (mean, sd, missing)	6.3 (1.8), n=52		
Relationship with care recipient	Relationship quality (mean, sd, missing)	23.4 (4.7), n=22	22.5 (4.9), n=43	22.2 (4.8), n=32
CARE RECIPIENT				
Care recipient diagnosis (n, %)	AD	564 (56.7%)	442 (56.7%)	348 (57.9%)
	VaD	103 (10.4%)	70 (9.0%)	55 (9.2%)
	Mixed AD/VaD	192 (19.3%)	164 (21.0%)	119 (19.8%)
	FTD	41 (4.1%)	34 (4.4%)	28 (4.7%)
	PDD/DLB	68 (6.8%)	53 (6.8%)	37 (6.2%)
	Unspecified/Other	27 (2.7%)	17 (2.2%)	14 (2.3%)
Care recipient measures	ACE-III cognition total score (mean, sd, missing)	69.4 (13.7), n=64	66.1 (16.3), n=60	64.1 (18.4), n=103
	Dependence (informant) (mean, sd, missing)	5.5 (2.6), n=64	6.2 (2.9), n=36	6.9 (3.0), n=32
	Functional ability (informant) (mean, sd, missing)	17.3 (8.5), n=72	20.3 (8.5), n=39	22.4 (8.6), n=21
	NP symptoms – caregiver distress (mean, sd, missing)	7.0 (6.3), n=285	6.8 (6.9), n=117	7.4 (6.9), n=79

Note. Alzheimer’s disease, AD; vascular dementia, VaD; frontotemporal dementia, FTD; Parkinson’s disease dementia, PDD; dementia with Lewy bodies, DLB; neuropsychiatric, NP; World Health Organization Quality of Life, WHOQOL; Satisfaction with Life Scale, SwLS; WHO-5, World Health Organization-Five Well-being Index; Addenbrooke’s Cognitive Examination III, ACE-III; standard deviation, sd.

Supplementary Table 2. Testing factorial measurement invariance for the 'living well' latent factor for spouse caregivers

**Model**	**BIC**	**X^2(df)**	**CFI**	**TLI**	**RMSEA (90% CI)**	**SRMR**	**ΔX^2(Δdf)/p**	**ΔCFI**	**ΔTLI**	**ΔRMSEA**	**ΔSRMR**
Configural	39452	26.97(16)	0.998	0.996	0.026 (0.005 – 0.043)	0.016					
Metric	39434	36.35(20)	0.997	0.995	0.029 (0.013 – 0.043)	0.028	9.38(4)/0.052	0.001	0.001	0.003	0.012
Scalar	39449	79.79(24)	0.990	0.986	0.043 (0.031 – 0.054)	0.036	43.44(4)/<0.001	0.007	0.008	0.014	0.008
Strict	39413	84.40(30)	0.991	0.982	0.043 (0.032 – 0.054)	0.033	4.61(6)/0.595	0.001	0.004	0.000	0.003

Note. The configural model has no constraints. The metric model has constrained factor loadings across each measurement occasion. The scalar model has constrained factor loadings across each measurement occasion and intercepts across time are set to be equal. The strict model has constrained factor loadings across measurement occasions, and intercepts and variances across time are set to be equal. ΔCFI, ΔRMSEA, and ΔSRMR were determined to assess model fit. ΔX^2 was examined but not relied upon.

Bayesian Information Criterion, BIC; degrees of freedom, df; comparative fit index, CFI; Tucker-Lewis index, TLI; root mean square error of approximation, RMSEA; standardized root mean squared residual, SRMR.

Supplementary Table 3. Testing increasing numbers of classes in LCGA and GMM models for 'living well' of spouse caregivers

	1 class	2 class	3 class	4 class	5 class
LCGA					
LL	-20235.5	-19770.5	-19655.3	-19618.5	-19610.4
BIC	40616.0	39706.7	39497.1	39444.1	39448.7
SSABIC	40549.3	41317.9	39411.3	39348.9	39343.9
Entropy	1	0.730	0.700	0.674	0.690
Adj MLR-LRT (p)	-	887.3 (0.000)	219.7 (0.001)	70.3 (0.000)	15.4 (0.050)
BLRT (p)	-	930.1 (0.000)	230.3 (0.000)	73.7 (0.000)	16.2 (0.000)
Group size (%)					
C1	100	56.3	33.6	24.1	24.1
C2	-	43.7	47.1	38.8	38.4
C3	-	-	19.3	29.1	3.4
C4	-	-	-	8.0	26.4
C5	-	-	-	-	7.7
GMM-CI					
LL	-19623.4	-19612.3	-19604.0	-19597.6	-19595.5
BIC	39412.5	39411.0	39415.2	39423.2	39439.7
SSABIC	39336.3	39325.3	39319.9	39318.4	39325.3
Entropy	1	0.521	0.581	0.598	0.544
Adj MLR-LRT (p)	-	21.2 (0.001)	15.80 (0.051)	12.2 (0.558)	4.0 (0.423)
BLRT (p)	-	22.2 (0.000)	16.56 (0.000)	12.7 (0.050)	4.2 (0.667)
Group size (%)					
C1	100	72.6	66.8	62.1	38.3
C2	-	27.4	26.0	25.5	10.4
C3	-	-	7.2	9.9	10.5
C4	-	-	-	2.6	3.3
C5	-	-	-	-	37.5

Note. In the LCGA, variances and covariances are set to zero. In the GMM-CI, variances and covariances are fixed between classes for the intercept and slope. No solutions are presented for the full freed GMM-CV model, due to inadmissible solutions.

Latent class growth analysis, LCGA; growth mixture modelling – class invariant, GMM-CI; log-likelihood, LL; Bayesian Information Criterion, BIC; samples size adjusted BIC; ssaBIC; Lo-Mendell-Rubin adjusted Likelihood Ratio Test, adj LMR-LRT; Bootstrap Likelihood Ratio Test, BLRT; class 1-class 5, C1-C5.

Supplementary Table 4. Average latent class probabilities for most likely latent class membership (row) by latent class (column) for the 3-class model of ‘living well’ for spouse caregivers

	**Class 1. Stable**	**Class 2. Lower Stable**	**Class 3. Declining**
Class 1. Stable	0.828	0.114	0.059
Class 2. Lower Stable	0.228	0.772	0.006
Class 3. Declining	0.222	0.001	0.771

Note. Each column represents classification probabilities (averages of the individual probabilities in each class) for the 3-class solution of the estimated GMM-CI, and each row represents the classification probabilities for the most likely class. If classes were perfectly separated, the diagonal components would be 1, and the off diagonals would be 0.

Supplementary Figure 2. Plots of the LCGA and GMM-CI models for ‘living well’ associated with the models presented in Supplementary Table 3

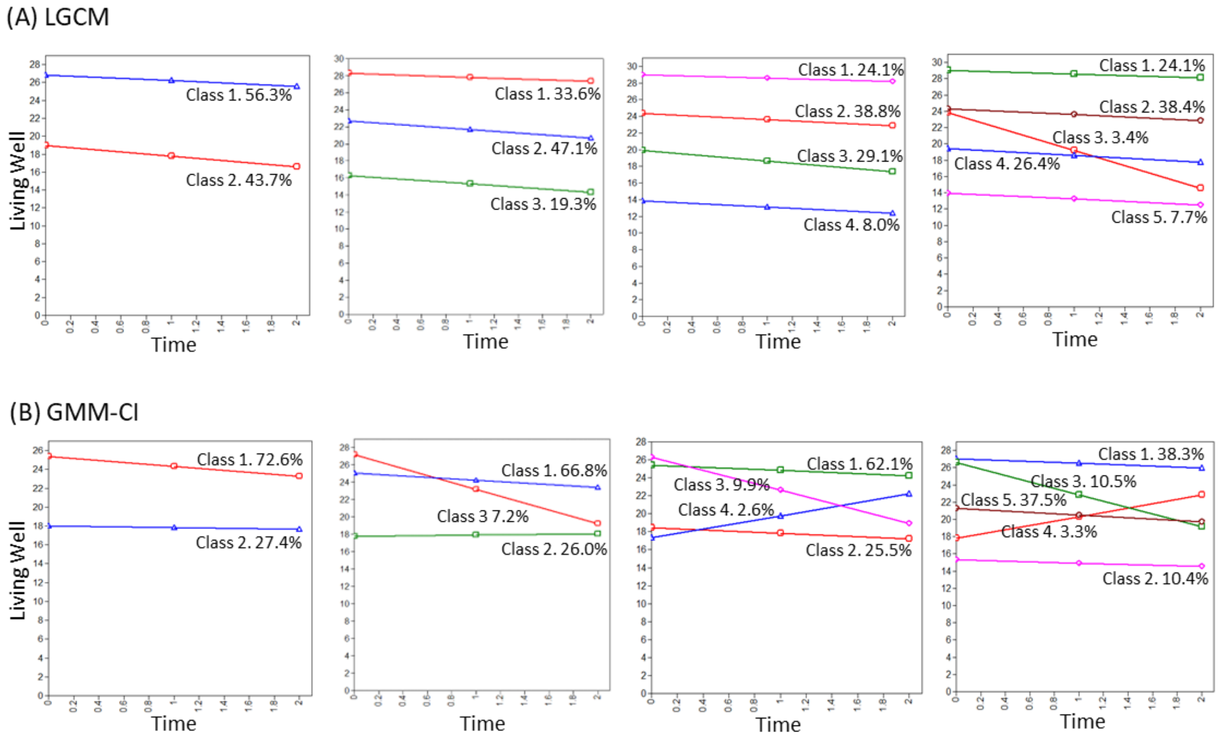

Supplementary Figure 3. Plots of individual spouse caregivers, excluding those who are caregivers of someone in a care home, within the three classes of trajectories of ‘living well’. The most likely class is allocated based on posterior probabilities for graphing purposes. For the Stable and Lower Stable classes, a random sample of 100 caregivers was selected.

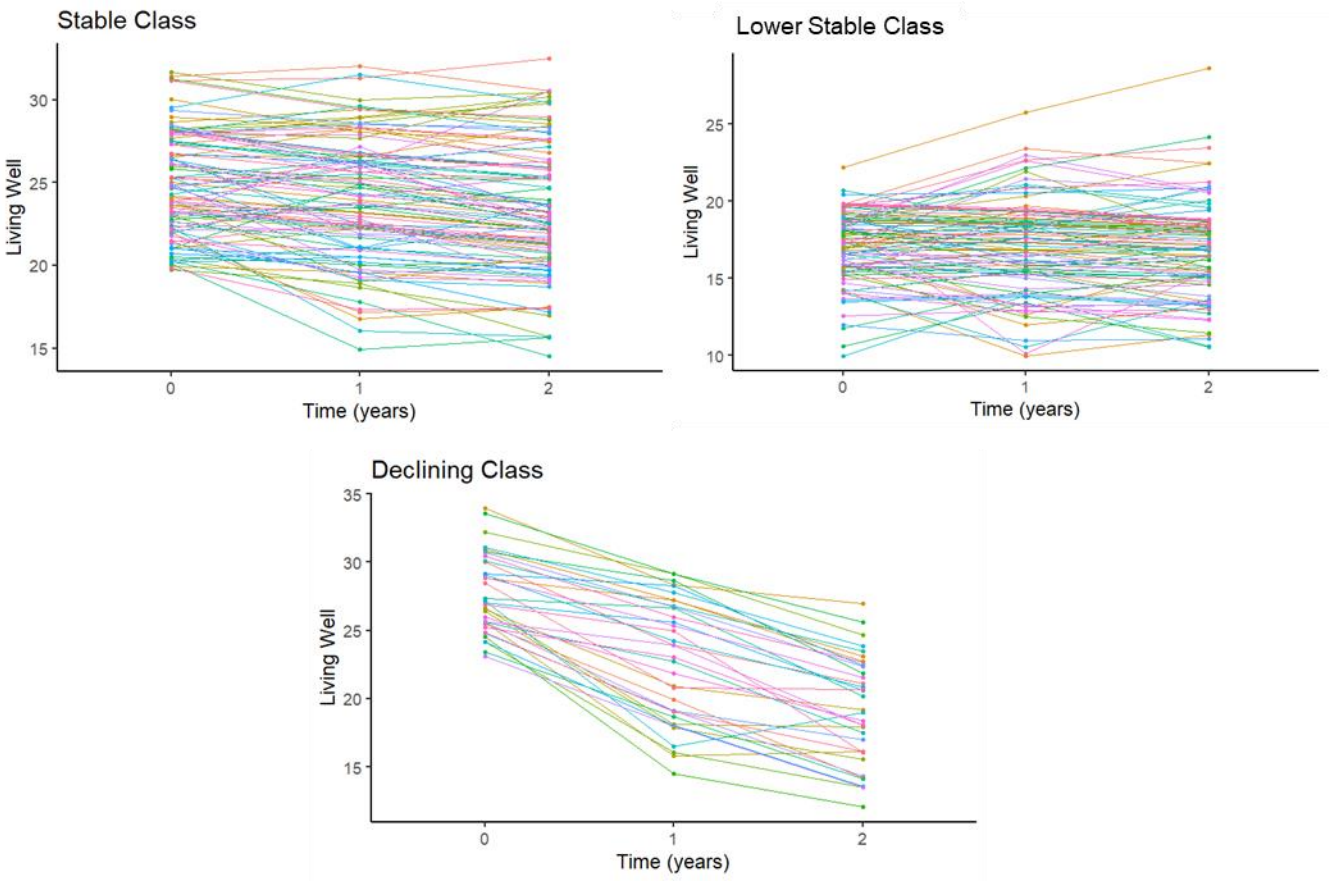

Supplementary Table 5. Sensitivity analysis comparing the missing at random model with models that account for non-ignorable dropout; Roy latent dropout pattern mixture model and Beunckens selection model

	Missing at random model			Roy pattern mixture model			Beunckens Selection model		
**Measure**	**%**	**Intercept**	**Slope**	**%**	**Intercept**	**Slope**	**%**	**Intercept**	**Slope**
SwLS									
Class 1	10.2	12.36 (10.76 - 13.97)	0.87 (0.04 - 1.69)	9.9	12.27 (10.62 - 13.91)	0.83 (0.04 - 1.63)	10.0	12.25 (10.56 - 13.94)	0.84 (0.05 - 1.63)
Class 2	35.2	19.68 (18.72 - 20.63)	-0.63 (-1.10 - -0.16)	35.4	19.62 (18.67 - 20.58)	-0.60 (-1.06 - 0.14)	35.4	19.62 (18.64 - 20.60)	-0.60 (-1.10 - -0.10)
Class 3	54.5	28.53 (28.13 - 28.93)	-2.12 (-2.45 - -1.79)	54.7	28.52 (28.12 -28.92)	-2.10 (-2.43 - -1.78)	54.6	28.52 (28.12 - 28.92)	-2.10 (-2.46 - -1.75)
WHO-5									
Class 1	30.4	33.75 (30.94 - 36.57)	2.83 (1.07 - 4.58)	30.5	33.75 (30.87 - 36.63)	2.93 (1.17 - 4.69)	30.4	33.65 (30.79 - 36.50)	2.71 (0.85 - 4.57)
Class 2	47.5	67.78 (65.65 - 69.91)	-1.29 (-2.33 - -0.26)	46.2	68.27 (66.06 - 70.12)	-1.50 (-2.60 - -0.40)	47.5	68.10 (65.90 - 70.31)	-1.52 (-2.60 - -0.45)
Class 3	22.2	58.06 (53.58 - 62.53)	-12.72 (-15.70 - -9.74)	23.3	58.72 (54.00 - 63.44)	-12.84 (15.95 - -9.73)	22.2	58.19 (53.57 - 92.81)	-12.94 (16.00 - -9.88)

Note. Satisfaction with Life Scale, SwLS; WHO-5, World Health Organization-Five Well-being Index.

Supplementary Table 6. Characteristics of spouse caregivers and care recipients, and baseline scores on study variables, for each latent ‘living well’ class

(a) Characteristics and scores on measures available at baseline (T1) only

Domain	Measure	Class 1. Stable (n=665, 66.8%)	Class 2. Lower Stable (n=259, 26.0%)	Class 3. Declining (n=72, 7.2%)
CAREGIVER				
Characteristics	Caregiver/care recipient sex (n, %)			
	Female/male	413 (62.1%)	201 (77.6%)	42 (58.3%)
	Male/female	247 (37.1%)	57 (22.0%)	29 (40.3%)
	Female/female	4 (0.6%)	1 (0.4%)	1 (1.4%)
	Male/male	1 (0.1%)	0 (0.0%)	0 (0.0%)
	Caregiver education (n, %)			
	No qualifications	155 (23.6%)	78 (30.5%)	16 (22.5%)
	School leaving certificate at 16	143 (21.8%)	65 (25.4%)	14 (19.7%)
	School leaving certificate at 18	199 (30.3%)	67 (26.2%)	24 (33.8%)
	University	160 (24.4%)	46 (18.0%)	17 (23.9%)
	Caregiver social class (n, %)			
	High	302 (50.0%)	103 (44.6%)	35 (53.8%)
	Middle	257 (42.5%)	102 (44.2%)	26 (40.0%)
	Low	45 (7.5%)	26 (11.3%)	4 (6.2%)
Psychological health	Neuroticism (mean, sd)	10.2 (2.9)	12.7 (2.9)	9.8 (2.8)
	Self-esteem (mean, sd)	32.1 (3.9)	28.5 (3.9)	32.7 (4.6)
	Self-efficacy (mean, sd)	32.1 (4.0)	30.1 (4.0)	33.3 (4.3)
	Optimism (mean, sd)	15.3 (3.1)	12.6 (3.4)	15.9 (3.5)
Physical health	Self-rated health (mean, sd)	4.2 (1.0)	3.5 (1.0)	4.3 (1.0)
	Health conditions (mean, sd)	5.3 (2.5)	5.8 (2.9)	5.4 (2.3)

Social situation	Social comparison (mean, sd)	3.5 (0.9)	2.9 (0.9)	3.7 (0.9)
	Standing in society (mean, sd)	6.8 (1.4)	6.1 (1.5)	6.8 (1.6)
	Standing in the community (mean, sd)	6.5 (1.7)	5.7 (1.8)	6.5 (1.7)
	Frequency of social contact (mean, sd)	19.5 (4.8)	18.6 (4.9)	19.8 (5.0)
	Cultural activity (mean, sd)	25.0 (5.3)	23.6 (5.2)	25.1 (5.6)
	Civic participation (n, %)			
	No participation	442 (70.0%)	168 (68.3%)	50 (73.5%)
	Low participation	118 (18.7%)	54 (22.0%)	10 (14.7%)
	High participation	71 (11.3%)	24 (9.8%)	8 (11.8%)
	Social participation (n, %)			
	No participation	342 (54.6%)	137 (56.4%)	36 (52.2%)
	Low participation	102 (16.3%)	40 (16.5%)	9 (13.0%)
	High participation	182 (29.1%)	66 (27.2%)	24 (34.8%)
Relationship with care recipient	Relationship quality (mean, sd)	24.2 (4.3)	20.8 (5.0)	24.9 (4.2)
CARE RECIPIENT				
Care recipient diagnosis (n, %)	AD	373 (56.1%)	139 (53.5%)	43 (60.0%)
	VaD	69 (10.4%)	27 (10.3%)	5 (7.5%)
	Mixed AD/VaD	132 (19.8%)	50 (19.3%)	15 (21.2%)
	FTD	30 (4.5%)	11 (4.4%)	2 (3.2%)
	PDD/DLB	45 (6.8%)	26 (10.1%)	5 (6.6%)
	Unspecified/Other	16 (2.4%)	6 (2.5%)	1 (1.4%)

(b) Scores on measures available longitudinally

Domain	Measure	Class 1. Stable (n=665, 66.8%)			Class 2. Lower Stable (n=259, 26.0%)			Class 3. Declining (n=72, 7.2%)		
		T1	T2	T3	T1	T2	T3	T1	T2	T3
CAREGIVER										
'Living well'	WHOQOL-BREF (mean, sd)	0.72 (1.55)	0.49 (1.71)	0.30 (1.9)	-1.97 (1.66)	-1.88 (1.96)	-1.79 (2.01)	1.55 (1.54)	0.11 (1.88)	-1.13 (1.85)
	WHO-5 (mean, sd)	61.1 (16.4)	59.5 (18.1)	57.6 (18.6)	36.8 (16.4)	37.9 (17.8)	39.8 (18.6)	68.2 (15.2)	57.4 (19.1)	43.9 (20.2)
	SwLS (mean, sd)	25.4 (5.5)	23.9 (6.1)	23.2 (6.2)	18.6 (5.8)	17.6 (6.4)	18.1 (6.3)	27.2 (5.3)	22.4 (7.0)	18.5 (6.3)
Characteristics	Caregiver age (mean, sd)	72.7 (8.2)	73.6 (7.9)	74.1 (7.9)	71.1 (8.7)	71.9 (8.4)	72.3 (8.4)	73.1 (7.5)	74.1 (7.2)	74.5 (7.0)
	Hours of care provided per day (n, %)									
	Under 1 hour	153 (23.6%)	93 (18.1%)	52 (12.7%)	32 (12.6%)	18 (9.7%)	11 (8.0%)	18 (25.7%)	4 (7.3%)	2 (4.3%)
	1-10 hours	240 (37.0%)	184 (35.7%)	152 (37.3%)	86 (33.9%)	61 (33.0%)	48 (34.8%)	28 (40.0%)	24 (43.6%)	16 (34.8%)
	10+ hours	256 (39.4%)	238 (46.2%)	204 (50.0%)	136 (53.5%)	106 (57.3%)	79 (57.2%)	24 (34.3%)	27 (49.1%)	28 (60.9%)
Psychological health	Loneliness (mean, sd)	2.1 (1.8)	-	2.6 (1.8)	3.6 (1.8)	-	3.7 (1.8)	1.9 (1.8)	-	3.3 (2.0)
	Depression (mean, sd)	4.9 (5.3)	6.0 (6.8)	6.8 (7.5)	12.9 (9.7)	14.2 (10.7)	13.9 (11.0)	4.5 (5.9)	7.7 (9.0)	10.8 (10.5)
Physical health	Self-rated health (mean, sd)	4.2 (1.0)	4.1 (1.0)	4.0 (1.0)	3.5 (1.0)	3.2 (1.0)	3.3 (1.0)	4.3 (1.0)	4.1 (1.0)	3.6 (1.0)
	Health conditions (mean, sd)	5.3 (2.5)	5.1 (1.8)	5.1 (1.8)	5.8 (2.9)	5.4 (2.0)	5.5 (2.0)	5.4 (2.3)	5.3 (1.8)	5.6 (2.2)
Experiences of caregiving	Stress (mean, sd)	16.7 (8.6)	19.6 (9.5)	21.1 (9.9)	25.9 (9.4)	27.9 (9.1)	28.0 (9.3)	16.0 (8.7)	23.1 (10.0)	28.3 (9.2)
	Social restriction (mean, sd)	3.4 (1.3)	3.6 (1.4)	3.7 (1.4)	4.0 (1.4)	4.1 (1.4)	4.3 (1.4)	3.3 (1.3)	3.8 (1.4)	3.9 (1.4)
	Role captivity (mean, sd)	5.0 (2.0)	5.5 (2.3)	5.7 (2.4)	6.5 (2.5)	6.9 (2.7)	6.8 (2.6)	4.9 (1.9)	6.3 (2.7)	7.0 (2.7)
	Competence (mean, sd)	9.4 (1.6)	9.3 (1.5)	9.2 (1.5)	8.6 (1.7)	8.5 (1.6)	8.6 (1.6)	9.6 (1.7)	9.0 (1.7)	8.6 (1.6)
	Management of meaning (mean, sd)	25.8 (4.6)	26.2 (4.6)	26.0 (4.4)	25.9 (4.3)	26.3 (4.2)	25.6 (4.2)	26.2 (4.6)	26.9 (4.2)	26.6 (4.2)

Social situation	Social network (mean, sd)	18.2 (5.4)	17.6 (5.3)	17.5 (5.2)	16.2 (5.2)	16.1 (4.8)	15.5 (5.1)	18.6 (5.6)	17.6 (5.7)	16.9 (5.9)
Relationship with care recipient	Relationship quality (mean, sd)	24.2 (4.3)	22.3 (4.6)	23.0 (4.5)	20.8 (5.0)	20.0 (5.1)	20.1 (5.2)	24.9 (4.2)	22.7 (5.1)	21.4 (4.8)
CARE RECIPIENT										
Measures relating to the care recipient	ACE-III cognition total score (mean, sd)	69.3 (13.5)	66.0 (16.2)	63.5 (18.3)	70.3 (13.7)	67.9 (17.7)	67.9 (17.7)	68.0 (14.0)	63.7 (16.2)	60.3 (18.1)
	Dependence (informant) (mean, sd)	5.2 (2.6)	6.0 (2.8)	6.8 (3.0)	6.3 (2.5)	6.7 (2.9)	7.2 (2.9)	5.1 (2.6)	6.2 (3.0)	7.1 (3.0)
	Functional ability (informant) (mean, sd)	16.7 (8.6)	20.0 (8.6)	22.1 (8.6)	19.3 (8.2)	21.3 (8.2)	22.5 (8.2)	15.9 (8.7)	20.8 (8.8)	24.3 (8.9)
	NP symptoms – caregiver distress (mean, sd)	5.8 (5.3)	5.6 (5.9)	6.2 (6.1)	10.3 (7.5)	10.0 (7.8)	10.3 (8.0)	5.6 (5.7)	8.1 (8.4)	10.2 (7.9)

Note. Alzheimer’s disease, AD; vascular dementia, VaD; frontotemporal dementia, FTD; Parkinson’s disease dementia, PDD; dementia with Lewy bodies, DLB; neuropsychiatric, NP; World Health Organization quality of life, WHOQOL; Satisfaction with Life Scale, SwLS; WHO-5, World Health Organization-Five Well-being Index; Addenbrooke’s Cognitive Examination-III, ACE-III; standard deviation, sd. There were 996 caregivers who were assigned posterior probabilities for each class; this includes 984 caregivers from T1, 778 from T2 and 601 from T3.

Appendix 5. Analyses of data for all caregivers including other family members and friends as well as spouses and partners

Supplementary Table 7. Characteristics of all caregivers (spouse and family/friend) and care recipients at T1, T2, and T3, and scores on study variables

	**T1**	**T2**	**T3**
Caregiver took part for first time (n)	1203	21	4
Caregiver returned (n)	-	896	695
Caregiver did not take part at this timepoint (n)	25	15	-
Caregiver withdrew at this timepoint (n)	-	292	237
Living Well measures:			
WHOQOL Environment (mean, sd, missing)	76.1 (12.9), n=40	75.1 (12.8), N=47	74.4 (12.9), N=28
WHOQOL Physical (mean, sd, missing)	70.2 (18.8), n=39	69.3 (18.4), N=46	68.0 (18.1), N=31
WHOQOL Psychological (mean, sd, missing)	68.3 (14.2), n=41	66.7 (14.4), N=45	65.8 (15.1), N=28
WHOQOL Social relations (mean, sd, missing)	67.7 (17.2), n=44	66.8 (16.5), N=47	65.3 (17.3), N=30
WHOQOL Quality of Life (mean, sd, missing)	3.8 (0.8), n=32	3.8 (0.8), N=43	3.7 (0.8), N=27
WHOQOL Health (mean, sd, missing)	3.4 (1.0), n=31	3.5 (1.0), N=42	3.5 (1.0), N=27
WHOQOL factor score (mean, sd, missing)	0.16 (2.10), n=49	-0.02 (2.10), n=51	-0.19 (2.14), n=33
WHO-5 (mean, sd, missing)	55.6 (19.7), n=36	54.5 (20.5), N=44	52.6 (20.4), N=29
SwLS (mean, sd, missing)	23.8 (6.5), n=41	22.5 (6.8), N=49	21.9 (6.6), N=33
Characteristics:			
Caregiver status (n, %)			
Spouse/partner*	997 (82.9%)	782 (85.3%)	603 (86.3%)
Family/friend	206 (17.1%)	135 (14.7%)	96 (13.7%)
Son/daughter/stepchild/son-/daughter-in law	180 (87.4%)	118 (87.4%)	84 (87.5%)
Grandchild	1 (0.5%)	1 (0.7%)	0 (0.0%)
Brother/sister	8 (3.9%)	3 (2.2%)	3 (3.1%)
Nephew/niece	5 (2.4%)	3 (2.2%)	2 (2.1%)
Friend	12 (5.8%)	10 (7.4%)	7 (7.3%)
Living situation (n, %)			
All caregivers			
Lives with care recipient	1054 (87.6%)	822 (89.6%)	628 (89.8%)
Does not live with care recipient	149 (12.4%)	95 (10.4%)	71 (10.2%)
Spouse/partner caregivers			
Lives with care recipient (spouse/partner)	995 (99.8%)	780 (99.7%)	601 (99.7%)

Does not live with care recipient (spouse/partner)	2 (0.2%)	2 (0.3%)	2 (0.3%)
Family/friend caregivers			
Lives with care recipient (family/friend)	59 (28.6%)	42 (31.1%)	27 (28.1%)
Does not live with care recipient (family/friend)	147 (71.4%)	93 (68.9%)	69 (71.9%)
Diagnosis (n, %)			
AD	678 (56.4%)	517 (56.4%)	405 (57.9%)
VaD	132 (11.0%)	84 (9.2%)	64 (9.2%)
Mixed AD/VaD	245 (20.4%)	205 (22.4%)	147 (21.0%)
FTD	43 (3.6%)	35 (3.8%)	29 (4.1%)
PDD/DLB	76 (6.3%)	57 (6.2%)	39 (5.6%)
Unspecified/Other	29 (2.4%)	19 (2.1%)	15 (2.1%)
Caregiver age (years) (mean, sd, missing)	69.3 (11.0)	70.5 (10.5)	71.2 (10.3)
Caregiver sex/care recipient sex (n, %)			
Female/male	701 (58.3%)	538 (58.8%)	407 (58.2%)
Male/female	358 (29.8%)	280 (30.6%)	218 (31.2%)
Female/female	131 (10.9%)	90 (9.8%)	68 (9.7%)
Male/male	13 (1.1%)	7 (0.8%)	6 (0.9%)
Caregiver education (n, %)			
No qualifications	265 (22.0%)	195 (21.3%)	144 (20.6%)
School leaving certificate at 16	266 (22.1%)	205 (22.4%)	154 (22.0%)
School leaving certificate at 18	362 (30.1%)	263 (28.7%)	203 (29.0%)
University	303 (25.2%)	240 (26.2%)	187 (26.8%)
Missing	7 (0.6%)	14 (1.5%)	11 (1.6%)
Caregiver social class (n, %)			
High	530 (44.1%)	407 (44.4%)	316 (45.2%)
Middle	463 (38.5%)	351 (38.3%)	265 (37.9%)
Low	96 (8.0%)	64 (7.0%)	48 (6.9%)
Missing	114 (9.5%)	95 (10.4%)	70 (10.0%)
Caregiver hours per day (n, %)			
Under 1 hour	269 (22.4%)	148 (16.1%)	84 (12.0%)
1-10 hours	465 (38.7%)	347 (37.8%)	277 (39.6%)
10+ hours	457 (38.0%)	399 (43.5%)	330 (47.2%)
Missing	12 (1.0%)	23 (2.5%)	8 (1.1%)
Psychological health:			
Neuroticism (mean, sd, missing)	10.9 (3.1), n=38	-	-
Loneliness (mean, sd, missing)	2.4 (1.9), n=83	-	2.9 (1.9), n=40
Depression (mean, sd, missing)	7.1 (8.0), n=79	8.3 (9.1), n=79	8.8 (9.7), n=51
Self-esteem (mean, sd, missing)	31.2 (4.5), n=63	-	-
Self-efficacy (mean, sd, missing)	31.7 (4.2), n=56	-	-
Optimism (mean, sd, missing)	14.7 (3.7), n=47	-	-
Physical health:			
Self-rated health (mean, sd, missing)	4.1 (1.1), n=14	3.9 (1.1), n=39	3.9 (1.1), n=20
Health conditions (mean, sd, missing)	5.1 (2.8), n=123	4.8 (2.0), n=85	4.9 (2.1), n=54
Experiences of caregiving:			
Stress (mean, sd, missing)	18.9 (9.8), n=83	21.5 (10.1), n=60	23.2 (10.3), n=42

Social restriction (mean, sd, missing)	3.5 (1.4), n=50	3.7 (1.4), n=55	3.8 (1.4), n=32
Role captivity (mean, sd, missing)	5.5 (2.2), n=48	5.9 (2.5), n=52	6.1 (2.6), n=30
Competence (mean, sd, missing)	9.2 (1.7), n=45	9.1 (1.6), n=52	9.0 (1.6), n=29
Management of meaning (mean, sd, missing)	25.9 (4.4), n=70	26.3 (4.5), n=72	26.0 (4.3), n=46
Social situation:			
Social comparison (mean, sd, missing)	3.4 (1.0), n=19	-	-
Standing in society (mean, sd, missing)	6.6 (1.5), n=48	-	-
Standing in the community (mean, sd, missing)	6.2 (1.8), n=63	-	-
Frequency of social contact (mean, sd, missing)	19.5 (4.9), n=126	-	-
Social network (Lubben) (mean, sd, missing)	17.6 (5.5), n=48	17.2 (5.3), n=50	16.9 (5.3), n=36
Cultural activity (mean, sd, missing)	25.1 (5.5), n=85	-	-
Civic participation (n, %)			
No participation	789 (65.6%)	-	-
Low participation	224 (18.6%)	-	-
High participation	126 (10.5%)	-	-
Missing	64 (5.3%)	-	-
Social participation (n, %)			
No participation	624 (51.9%)	-	-
Low participation	174 (14.5%)	-	-
High participation	337 (28.0%)	-	-
Missing	68 (5.7%)	-	-
Relationship:			
Relationship quality (mean, sd, missing)	23.2 (4.7), n=33	22.4 (4.8), n=52	22.1 (4.8), n=34
Measures relating to the care recipient:			
ACE-III cognition total score (mean, sd, missing)	69.2 (13.4), n=81	66.1 (16.0), n=78	63.9 (18.2), n=118
Dependence (informant) (mean, sd, missing)	5.6 (2.6), n=80	6.2 (2.9), n=43	6.9 (3.0), n=34
Functional ability (informant) (mean, sd, missing)	17.6 (8.6), n=92	20.4 (8.5), n=43	22.4 (8.6), n=23
NP symptoms – caregiver distress (mean, sd, missing)	7.1 (6.3), n=348	6.6 (6.7), n=123	7.6 (7.1), n=90

Note. Alzheimer's disease, AD; vascular dementia, VaD; frontotemporal dementia, FTD; Parkinson's disease dementia, PDD; dementia with Lewy bodies, DLB; neuropsychiatric, NP; World Health Organization Quality of Life, WHOQOL; WHO-5, World Health Organization-Five Well-being Index; Satisfaction with Life Scale, SwLS; Addenbrooke's Cognitive Examination III, ACE-III; standard deviation, sd. *The two non-cohabiting partners excluded from the main analysis were included in the spouse/partner category for these supplementary analyses.

Supplementary Table 8. Latent growth curve modelling of 'living well' over time and the effects of covariates for all caregivers

	**Mean intercept (estimate, 95% CI)**	**Mean slope (estimate, 95% CI)**
Unconditional Model:		
Means	23.44 (23.09 – 23.80)*	-0.74 (-0.88 – -0.61)*
Variance	20.72 (17.54 – 23.90)*	1.15 (0.10 – 2.19)*
Characteristics:		
Caregiver status (ref: spouse/partner)		
Family/friend	2.10 (1.12 – 3.08)*	0.28 (-0.19 – 0.75)
Living situation (ref: co-resident with care recipient)		
Not co-resident with care recipient	2.30 (0.81 – 3.79)	0.05 (-0.67 – 0.65)
Diagnosis (ref: AD)		
VaD	-0.55 (-1.47 – 0.37)	0.36 (-0.05 – 0.78)
Mixed AD/VaD	-0.24 (-0.95 – 0.47)	0.06 (-0.24 – 0.37)
FTD	0.17 (-1.32 – 1.66)	0.15 (-0.48 – 0.78)
PDD/DLB	-1.40 (-2.50 – -0.30)*	0.19 (-0.33 – 0.72)
Unspecified/Other	-1.18 (-3.12 – 0.77)	0.49 (-0.35 – 1.32)
Caregiver age (years)	0.05 (0.01 – 0.08)*	-0.00 (-0.02 – 0.01)
Caregiver sex/care recipient sex (ref: female/male)		
Male/female	2.16 (1.49 – 2.83)*	0.09 (-0.20 – 0.37)
Female/female	0.46 (-0.94 – 1.86)	0.02 (-0.64 – 0.69)
Male/male	3.80 (0.77 – 6.83)*	0.22 (-1.23 – 1.66)
Caregiver education (ref: school leaving certificate at 18)		
No qualifications	-1.05 (-1.86 – -0.24)*	0.27 (-0.09 – 0.63)
School leaving certificate at 16	-0.78 (-1.58 – 0.02)	0.23 (-0.12 – 0.59)
University	0.51 (-0.26 – 1.28)	-0.04 (-0.37 – 0.30)
Caregiver social class (ref: high)		
Middle	-0.46 (-1.09 – 0.17)	0.16 (-0.12 – 0.43)
Low	-0.71 (-1.84 – 0.42)	0.40 (-1.11 – 0.91)
Caregiver hours per day (ref: 10+ hours)		
Under 1 hour	2.98 (2.21 – 3.75)*	-0.06 (-0.40 – 0.28)
1-10 hours	1.28 (0.61 – 1.94)*	-0.01 (-0.31 – 0.29)
Psychological health:		
Neuroticism	-0.96 (-1.04 – -0.87)*	0.06 (0.02 – 0.10)*
Loneliness	-1.44 (-1.59 – -1.30)*	0.07 (0.01 – 0.14)*
Depression	-0.42 (-0.46 – -0.39)*	0.03 (0.02 – 0.05)*
Self-esteem	0.70 (0.64 – 0.76)*	-0.07 (-0.09 – -0.04)*
Self-efficacy	0.51 (0.44 – 0.58)*	-0.06 (-0.09 – -0.03)*
Optimism	0.79 (0.72 – 0.86)*	-0.07 (-0.10 – -0.04)*
Physical health:		
Self-rated health	2.26 (2.02 – 2.51)*	-0.17 (-0.28 – -0.05)*
Health conditions	-0.32 (-0.44 – -0.20)*	0.03 (-0.02 – 0.09)

Experiences of caregiving:		
Stress	-0.34 (-0.37 – -0.31)*	0.00 (-0.02 – 0.01)
Social restriction	-1.05 (-1.26 – -0.84)*	-0.01 (-0.10 – 0.09)
Role captivity	-1.01 (-1.14 – -0.88)*	-0.02 (-0.08 – 0.05)
Competence	1.10 (0.94 – 1.27)	-0.04 (-0.12 – 0.04)
Management of meaning	0.01 (-0.06 – 0.08)	0.02 (-0.05 – 0.01)
Social situation		
Social comparison	2.43 (2.13 – 2.74)*	-0.15 (-0.29 – -0.00)*
Standing in society	1.08 (0.89 – 1.27)*	-0.11 (-0.20 – -0.03)*
Standing in the community	0.73 (0.57 – 0.90)*	-0.03 (-0.10 – 0.05)
Frequency of social contact	0.23 (0.16 – 0.29)*	-0.03 (-0.05 – 0.00)
Social network (Lubben)	0.28 (0.23 – 0.33)*	-0.03 (-0.05 – -0.01)*
Cultural activity	0.21 (0.13 – 0.26)*	-0.03 (-0.05 – -0.00)*
Civic participation (ref: no participation)		
Low participation	-0.78 (-1.53 – -0.04)*	-0.01 (-0.33 – 0.31)
High participation	0.13 (-0.82 – 1.07)	-0.32 (-0.74 – 0.10)
Social participation (ref: no participation)		
Low participation	-0.02 (-0.87 – 0.82)	-0.08 (-0.46 – 0.30)
High participation	1.07 (0.41 – 1.73)*	-0.16 (-0.44 – 0.13)
Relationship:		
Relationship quality	0.49 (0.43– 0.56)*	-0.01 (-0.04 – 0.02)
Care recipient measures:		
ACE-III cognition total score	0.02 (-0.00 – 0.05)	0.01 (0.00 – 0.02)*
Dependence (informant)	-0.52 (-0.64 – -0.41)*	-0.01 (-0.06 – 0.04)
Functional ability (informant)	-0.14 (-0.18 – -0.11)*	-0.01 (-0.03 – 0.01)
NP symptoms – caregiver distress	-0.37 (-0.42 – -0.33)*	0.03 (0.01 – 0.05)*

Note. Confidence intervals, CI; Alzheimer’s disease, AD; vascular dementia, VaD; Mixed Alzheimer’s and vascular, Mixed AD/VaD; Frontotemporal dementia, FTD; Parkinson’s disease dementia/Dementia with Lewy bodies, PDD/DLB; neuropsychiatric, NP; Addenbrooke’s Cognitive Examination-III, ACE-III. Models were adjusted for sex and age of the caregiver, caregiver status, and care recipient diagnosis. *Confidence intervals do not span 0.

Supplementary Figure 4. Trajectories of the 3 classes of 'living well' for all caregivers, determined from the GMM-CI model; Class 1: Stable, Class 2: Lower Stable, Class 3: Declining. The mean intercepts and slopes associated with each class are shown, as are the variances and covariances. 95% confidence intervals are displayed in brackets.

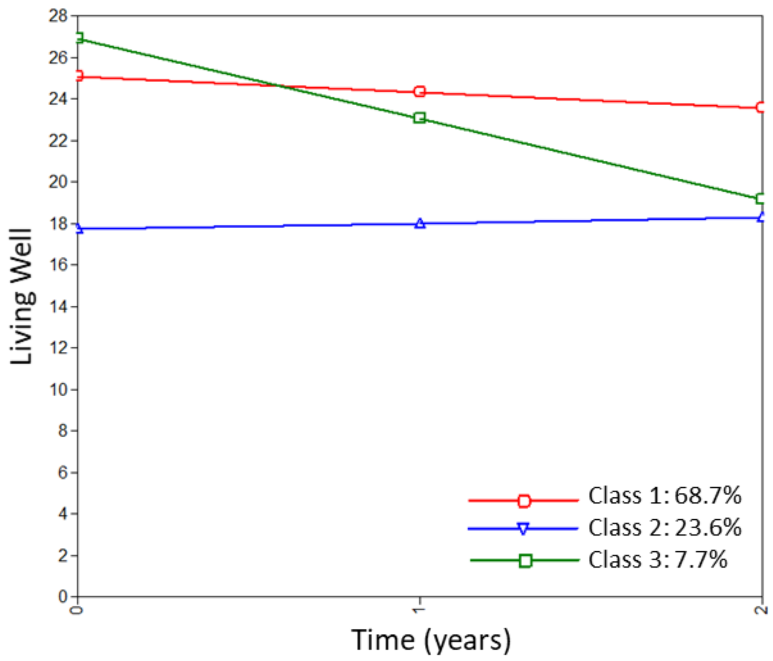

	**Class 1. Stable**	**Class 2. Lower Stable**	**Class 3. Declining**
Intercept	25.04 (24.05 – 26.03)*	17.71 (16.38 – 19.03)*	26.88 (25.39 – 28.38)*
Slope	-0.75 (-0.96 – -0.55)*	0.27 (-0.42 – 0.96)	-3.86 (-4.47 – -3.25)*
Variance-covariance			
Intercept	10.53 (7.12 – 13.93)	10.53 (7.12 – 13.93)	10.53 (7.12 – 13.93)
Slope	0.19 (-0.85 – 1.24)	0.19 (-0.85 – 1.24)	0.19 (-0.85 – 1.24)
Intercept-slope	2.03 (0.68 – 3.39)	2.03 (0.68 – 3.39)	2.03 (0.68 – 3.39)

Supplementary Figure 5. Plots of individual caregivers within the three classes of trajectories of 'living well' for all caregivers shown in Supplementary Figure 4. The most likely class is allocated based on posterior probabilities for graphing purposes. For the Stable and Lower Stable classes, a random sample of 100 caregivers were selected.

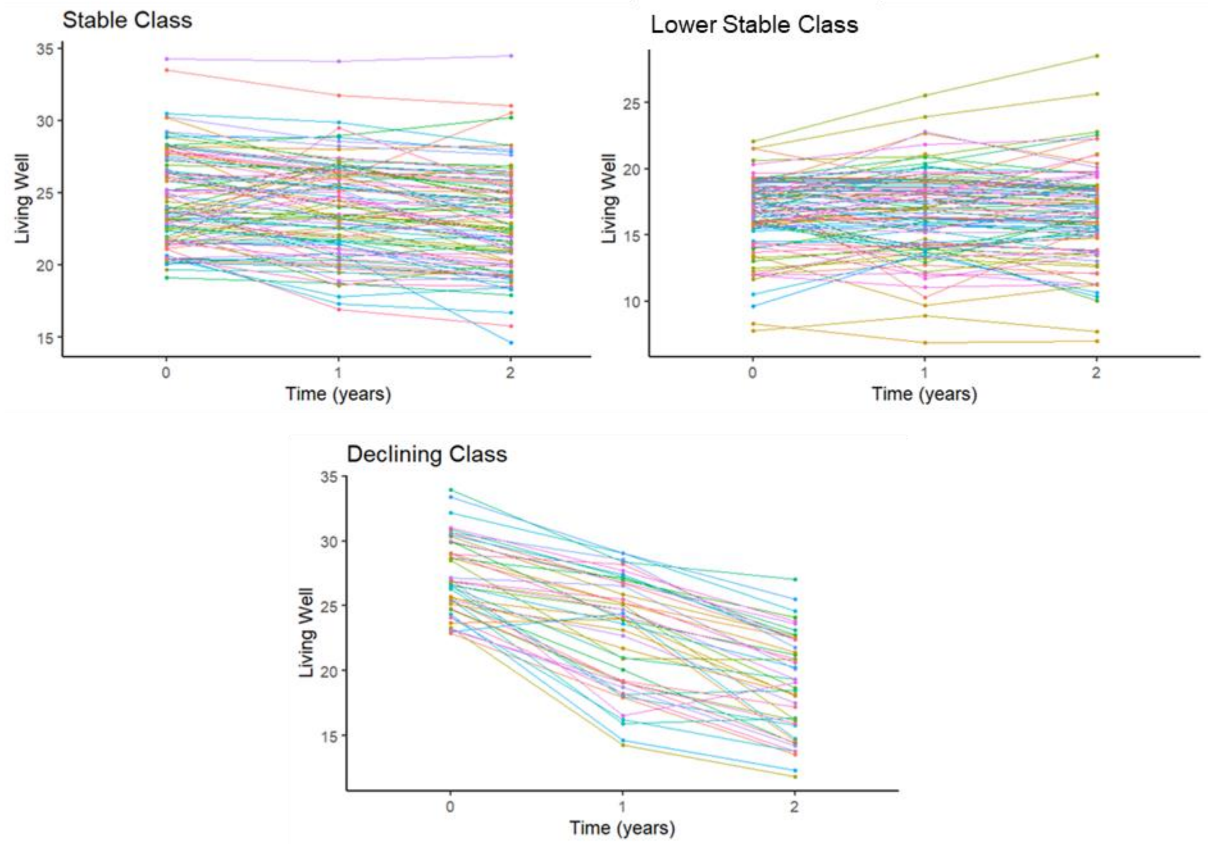

Supplementary Table 9. Characteristics of each latent 'living well' class for all caregivers

	**Class 1. Stable (n=829, 68.7%)**	**Class 2. Lower Stable (n=285, 23.6%)**	**Class 3. Declining (n=93, 7.7%)**
Characteristics:			
Caregiver status (n, %)			
Spouse/partner	684 (82.5%)	239 (83.6%)	75 (81.5%)
Family/friend	145 (17.5%)	47 (16.4%)	17 (18.5%)
Living situation (n, %)			
Co-resident with care recipient	720 (86.8%)	254 (89.2%)	80 (86.0%)
Not co-resident with care recipient	109 (13.2%)	31 (10.8%)	13 (14.0%)
Diagnosis (n, %)			
AD	462 (55.7%)	152 (53.3%)	57 (61.6%)
VaD	89 (10.7%)	32 (11.2%)	7 (7.7%)
Mixed AD/VaD	175 (21.1%)	58 (20.4%)	18 (19.8%)
FTD	33 (4.0%)	11 (3.9%)	3 (3.3%)
PDD/DLB	53 (6.4%)	26 (9.1%)	5 (5.5%)
Unspecified/Other	17 (2.1%)	6 (2.1%)	1 (1.1%)
Caregiver age (years) (mean, sd)	69.6 (10.8)	68.2 (11.0)	69.2 (11.5)
Caregiver sex/care recipient sex (n, %)			
Female/male	459 (55.4%)	195 (68.2%)	49 (52.7%)
Male/female	269 (32.5%)	59 (20.6%)	32 (34.4%)
Female/female	91 (11.0%)	30 (10.5%)	11 (11.8%)
Male/male	9 (1.1%)	2 (0.7%)	1 (1.1%)
Caregiver education (n, %)			
No qualifications (n, %)	172 (21.1%)	76 (27.0%)	18 (19.8%)
School leaving certificate at 16	174 (21.3%)	73 (25.9%)	17 (18.7%)
School leaving certificate at 18	251 (30.8%)	77 (27.3%)	31 (34.1%)
University	219 (26.8%)	56 (19.9%)	25 (27.5%)
Caregiver social class (n, %)			
High	376 (50.3%)	112 (44.4%)	42 (51.2%)
Middle	312 (41.7%)	111 (44.0%)	35 (42.7%)
Low	60 (8.0%)	29 (11.5%)	5 (6.1%)

Caregiver hours per day (n, %)			
Under 1 hour	202 (25.1%)	40 (14.3%)	23 (25.8%)
1-10 hours	319 (39.6%)	102 (36.6%)	37 (41.6%)
10+ hours	284 (35.3%)	137 (49.1%)	29 (32.6%)
Psychological health:			
Neuroticism (mean, sd)	10.3 (3.0)	12.9 (2.9)	10.0 (3.0)
Loneliness (mean, sd)	2.1 (1.8)	3.5 (1.9)	1.9 (1.7)
Depression (mean, sd)	5.2 (5.8)	13.5 (10.2)	5.1 (7.2)
Self-esteem (mean, sd)	32.0 (4.1)	28.1 (4.1)	32.7 (4.6)
Self-efficacy (mean, sd)	32.1 (4.0)	39.9 (4.3)	33.1 (4.3)
Optimism (mean, sd)	15.3 (3.3)	12.3 (3.7)	15.9 (3.5)
Physical health:			
Self-rated health (mean, sd)	4.3 (1.0)	3.5 (1.1)	4.4 (1.0)
Health conditions (mean, sd)	5.0 (2.7)	5.4 (3.0)	5.0 (2.5)
Experiences of caregiving:			
Stress (mean, sd)	16.8 (8.8)	25.8 (9.8)	16.4 (8.7)
Social restriction (mean, sd)	3.3 (1.3)	3.9 (1.4)	3.3 (1.4)
Role captivity (mean, sd)	5.2 (2.1)	6.6 (2.5)	5.0 (1.9)
Competence (mean, sd)	9.4 (1.6)	8.6 (1.7)	9.4 (1.7)
Management of meaning (mean, sd)	25.8 (4.5)	25.8 (4.3)	26.3 (4.5)
Social situation:			
Social comparison (mean, sd)	3.5 (0.9)	2.9 (0.9)	3.7 (0.9)
Standing in society (mean, sd)	6.8 (1.4)	6.0 (1.6)	6.9 (1.5)
Standing in the community (mean, sd)	6.4 (1.7)	5.7 (1.8)	6.4 (1.7)
Frequency of social contact (mean, sd)	19.7 (4.9)	18.6 (4.8)	20.1 (4.9)
Social network (Lubben) (mean, sd)	18.2 (5.5)	15.8 (5.4)	18.8 (5.5)
Cultural activity (mean, sd)	25.5 (5.4)	23.9 (5.4)	25.6 (5.6)
Civic participation (n, %)			

No participation	543 (69.7%)	185 (68.5%)	61 (70.1%)
Low participation	149 (19.1%)	60 (22.2%)	15 (17.2%)
High participation	87 (11.2%)	25 (9.3%)	11 (12.6%)
Social participation (n, %)			
No participation	419 (54.0%)	158 (58.7%)	46 (52.9%)
Low participation	120 (15.5%)	42 (15.6%)	11 (12.6%)
High participation	237 (30.5%)	69 (25.7%)	30 (34.5%)
Relationship:			
Relationship quality (mean, sd)	23.9 (4.4)	20.9 (5.1)	24.4 (4.3)
Care recipient measures:			
ACE-III cognition total score (mean, sd)	69.3 (13.2)	69.8 (13.7)	68.2 (13.4)
Dependence (informant) (mean, sd)	5.3 (2.6)	6.3 (2.6)	5.2 (2.6)
Functional ability (informant) (mean, sd)	17.1 (8.6)	19.4 (8.4)	16.4 (8.6)
NP symptoms – caregiver distress (mean, sd)	6.1 (5.3)	10.3 (7.6)	5.6 (5.5)

Note. Alzheimer’s disease, AD; vascular dementia, VaD; frontotemporal dementia, FTD; Parkinson’s disease dementia, PDD; dementia with Lewy bodies, DLB; neuropsychiatric, NP; Addenbrooke’s Cognitive Examination-III, ACE-III; standard deviation, sd. Misclassification error derived from the posterior probabilities is taken into account and numbers within each class are rounded to the nearest integer. There were 1207 caregivers who were assigned posterior probabilities for each class; this included 1185 caregivers from T1, 913 from T2 and 699 from T3.

Supplementary Table 10. Predicting class membership for 'living well' of all caregivers using multinomial logistic regression, adjusted for caregiver sex, caregiver age, caregiver status and care recipient diagnosis

	**Class 2. Lower Stable OR (95% CI) Ref: Stable**	**Class 3. Declining OR (95% CI) Ref: Stable**
Characteristics:		
Caregiver status (ref: spouse/partner)		
Family/friend	0.31 (0.16 – 0.96)*	0.87 (0.11 – 1.81)
Living situation (ref: co-resident with care recipient)		
Not co-resident with care recipient	0.36 (0.09 – 1.47)	0.66 (0.08 – 5.63)
Care recipient diagnosis (ref: AD)		
VaD	0.90 (0.42 – 1.94)	0.03 (0.00 – 43.63)
Mixed AD/VaD	1.33 (0.74 – 2.40)	0.73 (0.21 – 2.62)
FTD	0.62 (0.20 – 1.96)	0.13 (0.00 – 87.64)
PDD/DLB	1.87 (0.80 – 4.38)	0.85 (0.16 – 4.55)
Unspecified/Other	2.46 (0.63 – 9.62)	NE
Caregiver age (years)	0.96 (0.93 – 0.99)*	0.95 (0.90 – 1.00)
Caregiver sex/care recipient sex (ref: female/male)		
Male/female	0.30 (0.14 – 0.62)*	1.02 (0.33 – 3.16)
Female/female	0.71 (0.22 – 2.21)	1.55 (0.10 – 25.04)
Male/male	0.40 (0.03 – 4.94)	NE
Caregiver education (ref: school leaving certificate at 18)		
No qualifications	2.84 (1.26 – 6.44)*	0.87 (0.14 – 5.53)
School leaving certificate at 16	2.29 (1.06 – 4.97)*	0.66 (0.09 – 4.99)
University	0.97 (0.43 – 2.16)	0.68 (0.23 – 1.98)
Caregiver social class (ref: high)		
Middle	1.16 (0.65 – 2.08)	1.08 (0.35 – 3.38)
Low	2.04 (0.86 – 4.86)	0.19 (0.01 – 23.05)
Caregiver hours per day (ref: 10+ hours)		
Under 1 hour	0.16 (0.06 – 0.43)*	1.68 (0.39 – 7.20)
1-10 hours	0.51 (0.29 – 0.91)*	2.03 (0.60 – 6.84)
Psychological health:		
Neuroticism	2.05 (1.75 – 2.39)*	1.07 (0.91 – 1.27)
Loneliness	2.91 (2.16 – 3.93)*	1.02 (0.66 – 1.57)
Depression	1.42 (1.29 – 1.58)*	1.17 (0.85 – 1.60)
Self-esteem	0.49 (0.38 – 0.65)*	1.06 (0.83 – 1.36)
Self-efficacy	0.69 (0.54 – 0.88)*	1.16 (0.98 – 1.38)
Optimism	0.51 (0.43 – 0.61)*	1.09 (0.91 – 1.31)
Physical health:		
Self-rated health	0.14 (0.08 – 0.23)*	0.85 (0.46 – 1.60)
Health conditions	1.22 (1.05 – 1.43)*	1.07 (0.83 – 1.37)

Experiences of caregiving:		
Stress	1.29 (1.22 – 1.36)*	1.02 (0.93 – 1.12)
Social restriction	1.86 (1.49 – 2.32)*	1.25 (0.80 – 1.96)
Role captivity	1.70 (1.48 – 1.97)*	0.95 (0.70 – 1.29)
Competence	0.52 (0.41 – 0.65)*	0.88 (0.58 – 1.32)
Management of meaning	1.01 (0.95 – 1.07)	1.10 (0.99 – 1.22)
Social situation:		
Social comparison	0.14 (0.08 – 0.25)*	1.40 (0.71 – 2.77)
Standing in society	0.45 (0.35 – 0.58)*	0.74 (0.46 – 1.18)
Standing in the community	0.59 (0.49 – 0.70)*	0.86 (0.64 – 1.16)
Frequency of social contact	0.88 (0.84 – 0.94)*	1.02 (0.90 – 1.15)
Social network (Lubben)	0.85 (0.80 – 0.90)*	1.01 (0.88 – 1.15)
Cultural activity	0.88 (0.83 – 0.93)*	0.96 (0.85 – 1.09)
Civic participation (ref: no participation)		
Low participation	1.41 (0.76 – 2.62)	0.63 (0.15 – 2.69)
High participation	0.84 (0.36 – 1.95)	1.76 (0.51 – 6.03)
Social participation (ref: no participation)		
Low participation	0.65 (0.29 – 1.44)	0.14 (0.01 – 7.43)
High participation	0.67 (0.37 – 1.23)	1.10 (0.34 – 3.55)
Relationship:		
Relationship quality	0.72 (0.65 – 0.79)*	1.01 (0.81 – 1.27)
Measures relating to the care recipient:		
ACE-III cognition total score	1.00 (0.98 – 1.02)	1.01 (0.97 – 1.04)
Dependence (informant)	1.38 (1.22 – 1.56)*	0.96 (0.81 – 1.13)
Functional ability (informant)	1.07 (1.03 – 1.11)*	0.97 (0.92 – 1.02)
NP symptoms – caregiver distress	1.30 (1.20 – 1.41)*	1.02 (0.86 – 1.22)

Note. Alzheimer’s disease, AD; vascular dementia, VaD; frontotemporal dementia, FTD; Parkinson’s disease dementia, PDD; dementia with Lewy bodies, DLB; neuropsychiatric, NP; Addenbrooke’s Cognitive Examination-III, ACE-III; standard deviation, sd; reference category, ref; not estimated, NE. Odds ratios and confidence intervals could not be estimated for some groups due to small sample size. *Confidence intervals do not span 1.

References

Adler, N. E., Epel, E. S., Castellazzo, G., & Ickovics, J. R. (2000). Relationship of subjective and objective social status with psychological and physiological functioning: preliminary data in healthy white women. *Health Psychology*, *19*(6), 586-592. https://doi.org/10.1037/0278-6133.19.6.586

Asparouhov, T., & Muthen, B. (2013). *Auxiliary variables in mixture modeling: a 3-step approach using Mplus. Mplus Web Notes: No. 15. Version 6*.

Balducci, C., Mnich, E., McKee, K. J., Lamura, G., Beckmann, A., Krevers, B., Wojszel, Z. B., Nolan, M., Prouskas, C., Bien, B., & Oberg, B. (2008). Negative impact and positive value in caregiving: validation of the COPE index in a six-country sample of carers. *The Gerontologist*, *48*(3), 276-286. https://doi.org/10.1093/geront/48.3.276

Bech, P. (2004). Measuring the dimension of psychological general well-being by the WHO-5. *Quality of Life Newsletter*, *32*(1), 15-16.

Bengtson, V. L., & Schrader, S. S. (1982). Parent-child relations. In D. J. Mangon & W. A. Peterson (Eds.), *Research instruments in social gerontology: social roles and social participation* (Vol. 2, pp. 115-185). University of Minnesota Press.

Beunckens, C., Molenberghs, G., Verbeke, G., & Mallinckrodt, C. (2008). A latent-class mixture model for incomplete longitudinal gaussian data. *Biometrics*, *64*(1), 96-105. http://www.jstor.org/stable/25502025

Bowling, A. (2005). Just one question: if one question works, why ask several? *Journal of Epidemiology and Community Health*, *59*(5), 342-345. https://doi.org/10.1136/jech.2004.021204

Brickman, A. M., Riba, A., Bell, K., Marder, K., Albert, M., Brandt, J., & Stern, Y. (2002). Longitudinal assessment of patient dependence in Alzheimer disease. *Archives of Neurology*, *59*(8), 1304-1308. https://doi.org/10.1001/archneur.59.8.1304

Brown, T. A. (2006). *Confirmatory factor analysis for applied research*. The Guilford Press.

Byrne, B. M., & Watkins, D. (2003). The issue of measurement invariance revisited. *Journal of Cross-Cultural Psychology*, *34*(2), 155-175. https://doi.org/10.1177/0022022102250225

Charlson, M. E., Charlson, R. E., Peterson, J. C., Marinopoulos, S. S., Briggs, W. M., & Hollenberg, J. P. (2008). The Charlson comorbidity index is adapted to predict costs of chronic disease in primary care patients. *Journal of Clinical Epidemiology*, *61*(12), 1234-1240. https://doi.org/10.1016/j.jclinepi.2008.01.006

Charlson, M. E., Pompei, P., Ales, K. L., & MacKenzie, C. R. (1987). A new method of classifying prognostic comorbidity in longitudinal studies: development and validation. *Journal of Chronic Diseases*, *40*(5), 373-383. https://doi.org/10.1016/0021-9681(87)90171-8

Chen, F. F. (2007). Sensitivity of goodness of fit indexes to lack of measurement invariance. *Structural Equation Modeling*, *14*(3), 464-504. https://doi.org/10.1080/10705510701301834

Cheung, G. W., & Rensvold, R. B. (2002). Evaluating goodness-of-fit indexes for testing measurement invariance. *Structural Equation Modeling*, *9*(2), 233-255. https://doi.org/10.1207/S15328007SEM0902_5

Clare, L., Wu, Y.-T., Quinn, C., Jones, I. R., Victor, C. R., Nelis, S. M., Martyr, A., Litherland, R., Pickett, J. A., Hindle, J. V., Jones, R. W., Knapp, M., Kopelman, M. D., Morris, R. G., Rusted, J. M., Thom, J. M., Lamont, R. A., Henderson, C., Rippon, I., Hillman, A., Matthews, F. E., & On behalf of the IDEAL study team. (2019). A comprehensive model of factors associated with capability to "live well" for family caregivers of people living with mild-to-moderate dementia: findings from the IDEAL study. *Alzheimer Disease and Associated Disorders*, *33*(1), 29-35. https://doi.org/10.1097/WAD.0000000000000285

De Jong Gierveld, J., & Van Tilburg, T. (2010). The De Jong Gierveld short scales for emotional and social loneliness: tested on data from 7 countries in the UN generations and gender surveys. *European Journal of Ageing*, *7*(2), 121-130. https://doi.org/10.1007/s10433-010-0144-6

Diener, E., Emmons, R. A., Larsen, R. J., & Griffin, S. (1985). The Satisfaction with Life Scale. *Journal of Personality Assessment*, *49*(1), 71-75. https://doi.org/10.1207/s15327752jpa4901_13

Donnellan, M. B., Oswald, F. L., Baird, B. M., & Lucas, R. E. (2006). The mini-IPIP scales: tiny-yet-effective measures of the Big Five factors of personality. *Psychological Assessment*, *18*(2), 192-203. https://doi.org/10.1037/1040-3590.18.2.192

Eaton, W. W., Smith, C., Ybarra, M., Muntaner, C., & Tien, A. (2004). Center for Epidemiologic Studies Depression Scale: review and revision (CESD and CESD-R). In M. E. Maruish (Ed.), *The use of psychological testing for treatment planning and outcomes assessment* (3rd ed., Vol. 3: Instruments for Adults, pp. 363-377). Lawrence Erlbaum.

Greene, J. G., Smith, R., Gardiner, M., & Timbury, G. C. (1982). Measuring behavioural disturbance of elderly demented patients in the community and its effects on relatives: a factor analytic study. *Age and Ageing*, *11*(2), 121-126. https://doi.org/10.1093/ageing/11.2.121

Grimm, K. J., & Ram, N. (2009). Non-linear growth models in Mplus and SAS. *Structural Equation Modeling*, *16*(4), 676-701. https://doi.org/10.1080/10705510903206055

Hipp, J. R., & Bauer, D. J. (2006). Local solutions in the estimation of growth mixture models. *Psychological Methods*, *11*(1), 36-53. https://doi.org/10.1037/1082-989X.11.1.36

Hsieh, S., Schubert, S., Hoon, C., Mioshi, E., & Hodges, J. R. (2013). Validation of the Addenbrooke's Cognitive Examination III in frontotemporal dementia and Alzheimer's disease. *Dementia and Geriatric Cognitive Disorders*, *36*(3-4), 242-250. https://doi.org/10.1159/000351671

Hu, L., & Bentler, P. M. (1999). Cutoff criteria for fit indexes in covariance structure analysis: Conventional criteria versus new alternatives. *Structural Equation Modeling*, *6*(1), 1-55. https://doi.org/10.1080/10705519909540118

Jung, T., & Wickrama, K. A. S. (2008). An introduction to latent class growth analysis and growth mixture modeling. *Social and Personality Psychology Compass*, *2*(1), 302-317. https://doi.org/10.1111/j.1751-9004.2007.00054.x

Kaufer, D. I., Cummings, J. L., Ketchel, P., Smith, V., MacMillan, A., Shelley, T., Lopez, O. L., & DeKosky, S. T. (2000). Validation of the NPI-Q, a brief clinical form of the Neuropsychiatric Inventory. *The Journal of Neuropsychiatry and Clinical Neurosciences*, *12*(2), 233-239. https://doi.org/10.1176/appi.neuropsych.12.2.233

Kline, R. B. (2011). *Principles and practice of structural equation modeling* (3rd ed.). The Guilford Press.

Little, R. J. A., & Rubin, D. B. (2002). *Statistical analysis with missing data* (2nd ed.). John Wiley and Sons. https://doi.org/10.1002/9781119013563

Little, T. D. (2013). *Longitudinal structural equation modelling*. The Guilford Press.

Lubben, J., Blozik, E., Gillmann, G., Iliffe, S., von Renteln Kruse, W., Beck, J. C., & Stuck, A. E. (2006). Performance of an abbreviated version of the Lubben Social Network Scale among three European community-dwelling older adult populations. *The Gerontologist*, *46*(4), 503-513. https://doi.org/10.1093/geront/46.4.503

Martyr, A., Clare, L., Nelis, S. M., Marková, I. S., Roth, I., Woods, R. T., Whitaker, C. J., & Morris, R. G. (2012). Verbal fluency and awareness of functional deficits in early-

stage dementia. *The Clinical Neuropsychologist*, *26*(3), 501-519. https://doi.org/10.1080/13854046.2012.665482

Millsap, R. E., & Cham, H. (2012). Investigating factorial invariance in longitudinal data. In B. Laursen, T. D. Little, & N. A. Card (Eds.), *Handbook of developmental research methods* (pp. 109-126). The Guilford Press.

Millsap, R. E., & Olivera-Aguilar, M. (2012). Investigating measurement invariance using confirmatory factor analysis. In R. H. Hoyle (Ed.), *Handbook of structural equation modeling* (pp. 380-392). The Guilford Press.

Morris, J. C., & National Alzheimer's Coordinating Center. (2008). *NACC Uniform Data Set (UDS) coding guidebook for initial visit packet*. National Institute on Aging, ADC Clinical Task Force, NACC, University of Washington.

Muthén, B. (2004). Latent variable analysis: growth mixture modeling and related techniques for longitudinal data. In D. Kaplan (Ed.), *Handbook of quantitative methodology for the social sciences*. Sage Publications. https://doi.org/10.4135/9781412986311.n19

Muthén, B., Asparouhov, T., Hunter, A. M., & Leuchter, A. F. (2011). Growth modeling with nonignorable dropout: alternative analyses of the STAR*D antidepressant trial. *Psychological Methods*, *16*(1), 17-33. https://doi.org/10.1037/a0022634

Muthén, B., & Shedden, K. (1999). Finite mixture modeling with mixture outcomes using the EM algorithm. *Biometrics*, *55*(2), 463-469. https://doi.org/10.1111/j.0006-341X.1999.00463.x

Muthén, L. K., & Muthén, B. O. (1998-2017). *Mplus User's Guide* (8th ed.). Muthén & Muthén.

Nylund, K. L., Asparouhov, T., & Muthen, B. (2007). Deciding on the number of classes in latent class analysis and growth mixture modeling: a Monte Carlo simulation study.

Structural Equation Modeling, *14*(4), 535-569. https://doi.org/10.1080/10705510701575396

Office for National Statistics. (2008). *Harmonised concepts and questions for social data sources, secondary standards. Social capital*. Office for National Statistics.

Office for National Statistics. (2010). *Standard occupational classification 2010. Volume 3. The national statistics socioeconomic classification: (Rebased on the SOC2010) User Manual*. Palgrave Macmillan.

Pearlin, L. I., Mullan, J. T., Semple, S. J., & Skaff, M. M. (1990). Caregiving and the stress process: an overview of concepts and their measures. *The Gerontologist*, *30*(5), 583-594. https://doi.org/10.1093/geront/30.5.583

Pfeffer, R. I., Kurosaki, T. T., Harrah, C. H., Jr., Chance, J. M., & Filos, S. (1982). Measurement of functional activities in older adults in the community. *Journal of Gerontology*, *37*(3), 323-329. https://doi.org/10.1093/geronj/37.3.323

Robertson, S. M., Zarit, S. H., Duncan, L. G., Rovine, M. J., & Femia, E. E. (2007). Family caregivers' patterns of positive and negative affect. *Family Relations*, *56*(1), 12-23. https://doi.org/10.1111/j.1741-3729.2007.00436.x

Rosenberg, M. (1965). *Society and the adolescent self-image*. Princeton University Press.

Roy, J. (2003). Modeling longitudinal data with nonignorable dropouts using a latent dropout class model. *Biometrics*, *59*(4), 829-836. https://doi.org/https://doi.org/10.1111/j.0006-341X.2003.00097.x

Scheier, M. F., Carver, C. S., & Bridges, M. W. (1994). Distinguishing optimism from neuroticism (and trait anxiety, self-mastery, and self-esteem): a reevaluation of the Life Orientation Test. *Journal of Personality and Social Psychology*, *67*(6), 1063-1078. https://doi.org/10.1037/0022-3514.67.6.1063

Schermelleh-Engel, K., Moosbrugger, H., & Müller, H. (2003). Evaluating the fit of structural equation models: tests of significance and descriptive goodness-of-fit measures. *Methods of Psychological Research*, *8*(2), 23-74.

Schwarzer, R., & Jerusalem, M. (1995). Generalized Self-Efficacy Scale. In J. Weinman, S. Wright, & M. Johnston (Eds.), *Measures in health psychology: a user's portfolio. causal and control beliefs* (pp. 35-37). NFER-NELSON.

Skevington, S. M., Lotfy, M., O'Connell, K. A., & WHOQOL Group. (2004). The World Health Organization's WHOQOL-BREF quality of life assessment: psychometric properties and results of the international field trial. A report from the WHOQOL group. *Quality of Life Research*, *13*(2), 299-310. https://doi.org/10.1023/B:QURE.0000018486.91360.00

Tein, J. Y., Coxe, S., & Cham, H. (2013). Statistical power to detect the correct number of classes in latent profile analysis. *Structural Equation Modeling*, *20*(4), 640-657. https://doi.org/10.1080/10705511.2013.824781

Thomson, K. (2004). *Cultural capital and social exclusion survey: technical report*. National Centre for Social Research.

Vermunt, J. K. (2010). Latent class modeling with covariates: two improved three-step approaches. *Political Analysis*, *18*(4), 450-469. https://doi.org/10.1093/pan/mpq025

Wickrama, K. A. S., Lee, T. K., O'Neal, C. W., & Lorenz, F. O. (2016). *Higher-order growth curves and mixture modeling with Mplus. A practical Guide*. Routledge. https://doi.org/10.4324/9781315642741

Wu, Y.-T., Clare, L., Matthews, F. E., & On behalf of the Improving the experience of Dementia and Enhancing Active Life (IDEAL) study team. (2021). Relationship between depressive symptoms and capability to live well in people with dementia and their carers: results from the Improving the experience of Dementia and Enhancing

Active Life (IDEAL) programme. *Aging & Mental Health*, *25*(1), 38-45. https://doi.org/10.1080/13607863.2019.1671316
